# Taurine reduces the risk for metabolic syndrome: a systematic review and meta-analysis of randomized controlled trials

**DOI:** 10.1038/s41387-024-00289-z

**Published:** 2024-05-16

**Authors:** Chih-Chen Tzang, Liang-Yun Chi, Long-Huei Lin, Ting-Yu Lin, Ke-Vin Chang, Wei-Ting Wu, Levent Özçakar

**Affiliations:** 1 School of Medicine, College of Medicine, National Taiwan University, Taipei, Taiwan, ROC; 2 School of Physical Therapy and Graduate Institute of Rehabilitation Science, College of Medicine, Chang Gung University, Linkou, Taoyuan, Taiwan, ROC; 3 Department of Physical Medicine and Rehabilitation, Lo-Hsu Medical Foundation, Inc., Lotung Poh-Ai Hospital, Yilan, Taiwan, ROC; 4 Department of Physical Medicine and Rehabilitation, National Taiwan University Hospital, College of Medicine, National Taiwan University, Taipei, Taiwan, ROC; 5 Department of Physical Medicine and Rehabilitation, National Taiwan University Hospital, Bei-Hu Branch, Taipei, Taiwan, ROC; 6 Center for Regional Anesthesia and Pain Medicine, Wang-Fang Hospital, Taipei Medical University, Taipei, Taiwan, ROC; 7 Department of Physical and Rehabilitation Medicine, Hacettepe University Medical School, Ankara, Turkey

**Keywords:** Nutrition, Metabolic syndrome

## Abstract

**Background:**

Metabolic syndrome (MetS) is a cluster of interconnected risk factors that significantly increase the likelihood of cardiovascular disease and type 2 diabetes. Taurine has emerged as a potential therapeutic agent for MetS. This meta-analysis of randomized controlled trials (RCTs) aimed to evaluate the effects of taurine supplementation on MetS-related parameters.

**Methods:**

We conducted electronic searches through databases like Embase, PubMed, Web of Science, Cochrane CENTRAL, and ClinicalTrials.gov, encompassing publications up to December 1, 2023. Our analysis focused on established MetS diagnostic criteria, including systolic blood pressure (SBP), diastolic blood pressure (DBP), fasting blood glucose (FBG), triglyceride (TG), and high-density lipoprotein cholesterol (HDL-C). Meta-regression explored potential dose-dependent relationships based on the total taurine dose administered during the treatment period. We also assessed secondary outcomes like body composition, lipid profile, and glycemic control.

**Results:**

Our analysis included 1024 participants from 25 RCTs. The daily dosage of taurine in the studies ranged from 0.5 g/day to 6 g/day, with follow-up periods varying between 5 and 365 days. Compared to control groups, taurine supplementation demonstrated statistically significant reductions in SBP (weighted mean difference [WMD] = −3.999 mmHg, 95% confidence interval [CI] = −7.293 to −0.706, *p* = 0.017), DBP (WMD = −1.509 mmHg, 95% CI = −2.479 to −0.539, *p* = 0.002), FBG (WMD: −5.882 mg/dL, 95% CI: −10.747 to −1.018, *p* = 0.018), TG (WMD: −18.315 mg/dL, 95% CI: −25.628 to −11.002, *p* < 0.001), but not in HDL-C (WMD: 0.644 mg/dl, 95% CI: −0.244 to 1.532, *p* = 0.155). Meta-regression analysis revealed a dose-dependent reduction in DBP (coefficient = −0.0108 mmHg per g, *p* = 0.0297) and FBG (coefficient = −0.0445 mg/dL per g, *p* = 0.0273). No significant adverse effects were observed compared to the control group.

**Conclusion:**

Taurine supplementation exhibits positive effects on multiple MetS-related factors, making it a potential dietary addition for individuals at risk of or already experiencing MetS. Future research may explore dose-optimization strategies and potential long-term benefits of taurine for MetS management.

## Introduction

Metabolic syndrome (MetS) poses a significant global health challenge, affecting over one billion people [1]. This cluster of interconnected risk factors is diagnosed by the presence of: (1) abdominal obesity with increased waist circumference, (2) high blood pressure, (3) high fasting blood glucose (FBG) levels, (4) high triglyceride (TG) levels, and (5) a low level of high-density lipoprotein cholesterol (HDL-C) [2]. MetS significantly increases the risk of various health problems, including cardiovascular disease, stroke, and type 2 diabetes [3]. Its development is associated with factors like insulin resistance, chronic inflammation, and neurohormonal activation [3].

Taurine (2-aminoethanesulfonic acid), a sulfur-containing amino acid, has gained scientific interest due to its potential to modulate various physiological processes. It is primarily obtained through diet, particularly from foods like shellfish, dark meat, and some energy drinks [4]. Abundant in tissues like the heart, retina, liver, muscle, and platelets, taurine plays crucial roles in osmoregulation, mitochondrial function, maintenance of cell membrane stability, antioxidative defense mechanisms, and regulating cation balance [4].

In addition to its fundamental functions, taurine shows promise for regulating key metabolic parameters associated with MetS. It plays a significant role in controlling lipid metabolism by conjugating with bile salts [4]. Studies have also suggested its potential to improve glycemic markers, including FBG, serum insulin levels, and glycated hemoglobin (HbA1c) [5]. Moreover, taurine may exert anti-inflammatory effects by inhibiting the renin-angiotensin system, a key factor in the development of cardiovascular diseases and obesity [6]. Therefore, taurine has the potential to positively affect MetS.

Despite numerous clinical studies demonstrating the diverse health benefits of taurine, inconsistencies in clinical outcomes make it challenging to definitely determine whether taurine reduces the risk of MetS [7, 8]. To address this knowledge gap, we conducted a meta-analysis of randomized controlled trials (RCTs) to systematically analyze the effects of taurine on modifying the parameters associated with MetS.

## Materials and methods

### General guidelines

This meta-analysis adhered to the most recent revision of Preferred Reporting Items for Systematic Reviews and Meta-Analyses 2020 guidelines (Table S1) [9]. The review was registered on Inplasy.com under number (INPLASY2023120081). Two authors (T.-C.C. and C.-L.Y.) conducted independent electronic searches in Embase, PubMed, Web of Science, Cochrane CENTRAL, and ClinicalTrials.gov databases using the following keywords (“taurine” OR “taufon”) AND (“metabolic syndrome” OR “diabetes mellitus” OR “obesity” OR “hypertension” OR “dyslipidemia” OR ‘hyperglycemia’). The search covered the inception of each database until December 1, 2023. The detailed search methodology is provided in the Supplementary Material (Table S2). The identified titles and abstracts were initially screened by the two authors to determine their eligibility, followed by a full-text review where necessary. We also manually searched additional databases and checked the reference lists of relevant meta-analyses. This study included publications in all languages.

### Inclusion and exclusion criteria

This meta-analysis followed the PICO (population, intervention, comparison, and outcome) settings design: P, human participants; I, taurine supplementation; C, supplementation (including placebo) other than taurine; and O, parameters associated with the diagnosis of MetS.

We included (1) RCTs employing pure taurine and its compounds as the treatment arm, (2) trials with a comparative arm using interventions other than taurine, and (3) studies providing data for pre- and post-intervention assessments of at least one outcome related to MetS.

We excluded (1) non-RCTs, including quasi-experimental studies such as real world observations, trials without a comparing placebo, retrospective studies, cohort studies and case reports; (2) studies with short follow-up periods unlikely to capture effects on MetS (e.g., less than 24 h); (3) trials using herbal treatments with unclear active ingredients; (4) studies lacking data for pre- and post-intervention endpoints; and (5) studies not investigating outcomes of interest.

Trials with follow-up durations less than 24 h and trials with unclear active ingredients were excluded as they did not align with the purpose of the study. Studies lasting less than 24 h primarily focus on the immediate effects of energy drinks, such as changes in heart rate, so we have opted to exclude them. Furthermore, studies that lacked precise quantification of active ingredients or failed to provide a comprehensive list of effective ingredients were excluded since we could accurately attribute observed effects solely to taurine.

### Methodological quality appraisal

The methodological quality of the included studies was assessed using the Cochrane risk of bias tool for RCTs (RoB 2, London, United Kingdom), which evaluates six main domains: randomization process, intervention adherence, missing outcome data, outcome measurement, selective reporting, and overall risk of bias [10]. Within the RoB 2 framework, intervention adherence can be assessed using two approaches: intention-to-treat and per-protocol. We opted for the per-protocol approach because most RCTs only report data from participants who completed the entire trial course [10].

### Primary and secondary outcome

The primary outcomes of this investigation were changes in (1) systolic blood pressure (SBP), (2) diastolic blood pressure (DBP), (3) FBG, (4) TG, and (5) HDL-C. The secondary outcomes included: (1) body composition measures like body weight (BW) and body mass index (BMI), (2) lipid profiles including total cholesterol (TC) and low-density lipoprotein cholesterol (LDL-C), (3) glycemic profiles including HbA1c, homeostatic model assessment (HOMA), and fasting insulin, and (4) adverse effects. To accommodate studies with no reported adverse events, the number of cells with zero events was adjusted to 0.5 [11].

### Data extraction and management

Two independent authors (T.-C.C. and C.-L.Y.) extracted data from the reviewed studies, including demographics, research design, details of taurine and control regimens, and outcome values. To avoid misinterpretation of effects, they carefully considered the direction of the scales used in each trial. In cases where data was missing from published studies, attempts were made to contact the corresponding authors to obtain the original data. Data extraction, conversion, and merging of outcomes from different study arms with varying taurine dosages were performed in accordance with the Cochrane Handbook for Systematic Review of Interventions and relevant medical literature [12–14]. For statistical analysis, the outcomes reported after the intervention were extracted, assuming data were available for multiple time points post-treatment.

### Statistical analyses

This meta-analysis was conducted using Comprehensive Meta-Analysis software (version 3; Biostat, Englewood, NJ, United States) due to the heterogeneous nature of the study populations [15]. For all continuous outcomes, the weighted mean difference (WMD) and its 95% confidence interval (CI) were calculated. Odds ratios and their corresponding 95% CIs were used to analyze categorical outcomes (i.e., the rates of treatment-related adverse events), such as the rates of treatment-related adverse events. The effects of outcomes were assessed using WMD, with respective units dependent on the variable. This metric illustrates the magnitude of change observed across the entire taurine intervention, regardless of dose and duration.

Heterogeneity between studies was assessed using I^2^ and Cochran’s Q statistics. I^2^ values of 25%, 50%, and 75% were considered indicative of low, moderate, and high heterogeneity, respectively [16]. To investigate potential dose-dependent relationships between taurine and primary outcomes, meta-regression analyses were conducted using the total taurine dose administered throughout the treatment period and the daily dosage. The total dose was calculated as the product of the duration of intake multiplied by the dosage per day. Thus, the coefficient represents the average effect per gram of administered taurine.

A one-study removal sensitivity analysis was conducted to assess whether excluding a specific trial significantly altered the overall effect size [11]. To investigate potential publication bias, we visually examined the distribution of effect sizes in a funnel plot and assessed the statistical significance of Egger’s regression test [9].

## Results

### Study selection

Our initial search yielded 2517 publications. After removing duplicates and screening titles and abstracts, we deemed 2476 articles irrelevant and discarded them. We then conducted a full-text review of the remaining 41 studies.

**Fig. 1:**
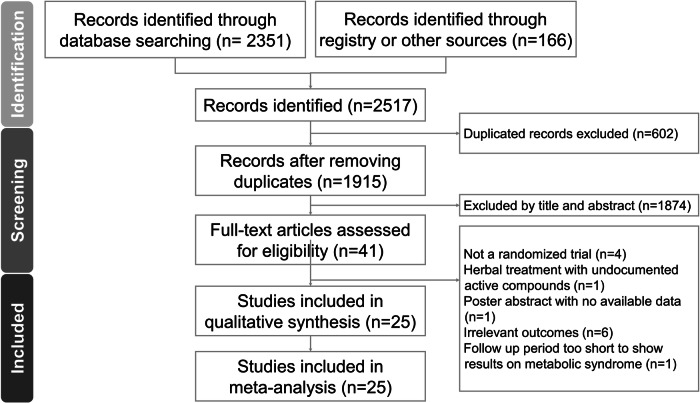
The PRISMA flow diagram of the screening and review process.

Thirteen articles were excluded for various reasons (Table S3): four weren’t RCTs, one used an herbal treatment with unverified active compounds, one was a poster abstract lacking data, six did not report outcomes aligned with our research focus, and one only administered a single dose of the intervention. This resulted in the inclusion of 25 studies [7, 8, 17–39] in our final quantitative analysis (Fig. 1). Data extraction details for these RCTs are presented in Tables 1 and 2.

**Table 1:** Summary of trials retrieved to investigate the impact of taurine on metabolic syndrome.

First author & year	Country	Population	Participants (F/M)	Age	Funding/grants/support
Azuma [22]	Japan	Congestive heart failure	58 (30/28)	38 - 89	N/A
Azuma [21]	Japan	Congestive heart failure	14 (5/9)	68.71 ± 9.10	Osaka University Medical School
Fujita [27]	Japan	Borderline hypertension	19 (N/A)	20 - 25	N/A
Azuma [23]	Japan	Idiopathic dilated cardiomyopathy	17 (6/11)	N/A	N/A
Mizushima [30]	Japan	Healthy male volunteers	20 (0/20)	18 - 29	N/A
Zhang [8]	Japan	Overweight or obese non-diabetic subjects	30 (16/14)	Taurine group 20.1 ± 2.0 Placebo group 20.4 ± 1.1	Taisho Pharmaceutical Co., Ltd., Japan
Spohr [7]	Denmark	Type 2 diabetes mellitus	18 (0/18)	40 ± 8	Steno Diabetes Center, Gentofte, Denmark, Aase and Ejnar Danielsens Foundation, Lyngby, Denmark
Moloney [31]	Ireland	Type 1 diabetes mellitus	19 (0/19)	28.0 ± 2.0	N/A
Beyranvand [25]	Iran	Heart failure with left ventricular ejection fraction less than 50%	29 (3/26)	60.57 ± 6.54	Shahid Beheshti Medical University
Arrieta [19]	Spain	Patients require amino-acid supplement after major surgical procedure	74 (21/53)	Taurine group: 48 – 90 Placebo group: 50 - 113	N/A
Hsieh [28]	China	Chronic alcoholic patients	30 (17/13)	Taurine group 59 ± 13 Placebo group 60 ± 12	Asia University (100-A-06), Chung Shan MedicalUniversity (CSMU-INT-102-12).
Rosa [34]	Brazil	Obese women	16 (16/0)	32 ± 2	National Council of Scientific and Technological Development (CNPq), State of Sao Paulo Research Foundation (FAPESP).
Averin [20]	Russia	Coronary heart disease/Heart valve defects	48 (12/36)	Taurine group: 49.79 ± 1.4 Placebo group 48.65 ± 1.5	N/A
Ra [33]	Japan	Healthy men	29 (0/29)	Taurine group: 25. 4 ± 1.0Placebo group: 25.2 ± 1.0	Japan Society for the Promotion of Science (JSPS)
Sun [37]	China	Prehypertensive individuals	86 (44/42)	56.75 ± 8.26	National Basic Research Program of China, National Natural Science Foundation of China
Ahmadian [17] a ^a^ (Journal of Dietary Supplements)	Iran	Heart failure	16 (N/A)	Taurine group: 60.12 ± 5.4 Placebo group: 60.13 ± 8.3	N/A
Ahmadian [18] b ^a^ (Ther Adv Cardiovasc Dis)	Iran	Heart failure	16 (N/A)	Taurine group: 60.12 ± 5.4 Placebo group: 60.13 ± 8.3	N/A
Schwarzer [35]	Austria	Patients with hepatic venous pressure gradient ≥12 mmHg	22 (8/14)	52 ± 11	N/A
Batitucci [24]	Brazil	Obese women	22 (22/0)	36.6 ± 6.4	Coordenação de Aperfeiçoamento de Pessoal de Nível Superior - Brasil (CAPES); The Sao Paulo Research Foundation (FAPESP)
Shari [36]	Iraq	Type 2 diabetes mellitus	80 (34/46)	Taurine group: 48.8 + 3.1 Placebo group: 50.2 ± 3.7	N/A
Van Hove [38]	USA	Inherited cystathionine -β-synthase deficient homocystinuria	36 (19/17)	8 - 50	Food and Drug Administration, Walter S. and Lucienne Driskill Foundation
Maleki [29]	Iran	Type 2 diabetes mellitus	45 (30/15)	Taurine group: 41.61 ± 6.63 Placebo group: 43.86 ± 7.06	Tabriz University of Medical Sciences, Tabriz, Iran
Esmaeili [26]	Canada	Type 2 diabetes mellitus	46 (32/14)^b^	Taurine group: 42.74 ± 7.21 Placebo group: 43.52 ± 6.94	N/A
Zaki [39]	Egypt	Peripartum cardiomyopathy	40 (40/0)	Taurine group: 31.1 ± 2.64 Placebo group: 30.85 ± 3.07	N/A
Moludi [32]	Iran	Type 2 diabetes mellitus	120 (97/23)	Taurine group: 52.13 ± 8.1 Placebo group: 53.08 ± 8.8	N/A

*AA* amino acid, *RCT* randomized controlled trial, *N/A* not applicable.

^a^Describes the same set of subjects, but provides different outcomes.

^b^Before loss of follow up.

**Table 2:** Summary of taurine interventions administered in the treatment arms of the trials.

First author & year	Daily Taurine dose (N)	Control (N)	Population	Duration	Taurine product/manufacturer
Azuma [22]	6 g/day (58)	Matching placebo (58)	Congestive heart failure	4 weeks^c^	Not mentioned
Azuma [21]	6 g/day (14)	Matching placebo (14)	Congestive heart failure	4 weeks^c^	Not mentioned
Fujita [27]	6 g/day (10)	Matching placebo (9)	Borderline hypertension	7 days	Not mentioned
Azuma [23]	3 g/day (7)	Active placebo (10)	Idiopathic dilated cardiomyopathy	6 weeks	Taurine sachet/Not mentioned
Mizushima [30]	6 g/day (11)	Matching placebo (9)	Healthy male volunteers	3 weeks	Taurine capsules/Taisho Pharmaceutical Co. Ltd.
Zhang [8]	3 g/day (15)	Comparable placebo (15)	Overweight or obese non-diabetic subjects	7 weeks	Taurine capsules/Jianli Pham Co., Beijing, China
Spohr [7]	1.5 g/day (18)	Matching placebo (44)	Type 2 diabetes mellitus	8 weeks^c^	Taurine capsules/Not mentioned
Moloney [31]	1.5 g/day (9)	Matching placebo (10)	Type 1 diabetes mellitus	14 days^c^	Taurine tablet/Twinlab
Beyranvand [25]	1.5 g/day (15)	Matching placebo (14)	Heart failure with left ventricular ejection fraction less than 50%	2 weeks	Taurine capsules/Solgar, Leonia, NJ, USA
Arrieta [19]	1.6 g/day^b^ (35)	Comparable placebo (39)	Patients require amino-acid supplement after major surgical procedure	24 days	Tauramin 10%,/Laboratorios Grifols SA, Barcelona, Spain
Hsieh [28]	6 g/day (15)	Matching placebo (15)	Chronic alcoholic patients	3 months	Taurine capsules/Dokui Chemical Company (Taiwan)
Rosa [34]	3 g/day (8)	Matching placebo (8)	Obese women	8 weeks	Taurine capsules/Ajinomoto CO., INC., Limeira, SP,Brazil
Averin [20]	0.5 g/day (24)	Matching placebo (24)	Coronary heart disease/Heart valve defects	3 months	Taurine capsules/Pik-Pharma, Russian Federation
Ra [33]	6 g/day (15)	Matching placebo (14)	Healthy men	15 days	Taurine capsules/Taisho Pharmaceutical Co., Ltd., Japan
Sun [37]	1.6 g/day (42)	Matching placebo (20)	Prehypertensive individuals	12 weeks	Taurine capsules/Not mentioned
Ahmadian [17]^a^ (Journal of Dietary Supplements)	1.5 g/day (8)	Matching placebo (8)	Heart failure	2 weeks	Taurine capsules/Solgar, Leonia, NJ, USA
Ahmadian [18]^a^ (Ther Adv Cardiovasc Dis)	1.5 g/day (8)	Matching placebo (8)	Heart failure	2 weeks	Taurine capsules/Solgar, Leonia, NJ, USA
Schwarzer [35]	6 g/day (12)	Matching placebo (10)	Patients with hepatic venous pressure gradient ≥12 mm Hg	4 weeks	Taurine capsules/Not mentioned
Batitucci [24]	3 g/day (14)	Matching placebo (8)	Obese women	8 weeks	Taurine powder/Aminoethylsulfonic Acid, Ajinomoto®, São Paulo, SP
Shari [36]	1 g/day (40)	Matching placebo (40)	Type 2 diabetes mellitus	3 months	Taurine capsules/Jarrow’s formulas
Van Hove [38]	5 g/day (14)	Matching placebo (22)	Inherited cystathionine -β-synthase deficient homocystinuria	5 days	Not mentioned
Maleki [29]	3 g/day (23)	Matching placebo (22)	Type 2 diabetes mellitus	8 weeks	Taurine capsules/Karen Pharma and Food Supplement Co., Iran,
Esmaeili [26]	3 g/day (23)	Matching placebo (23)	Type 2 diabetes mellitus	8 weeks	Taurine capsules/Karen Pharmaceutical Co
Zaki [39]	0.6 g/day (20)	Comparable placebo (18)	Peripartum cardiomyopathy	5 days	10 ml/kg taurine solution/10% (Aminoven®, Fresenius‑Kabi, Egypt)
Moludi [32]	3 g/day (60)	Matching placebo (60)	Type 2 diabetes mellitus	8 weeks	Taurine capsules/Karen Food Supplement Co, Iran

^a^Describes the same set of subjects, but provides different outcomes.

^b^1.5 g amino acid/kg bw/d, subjects are 69 kg in average.

^c^Treatment period of placebo or taurine in a crossover study.

### Study characteristic

Key features of the 25 RCTs, involving 1,024 participants, are summarized in Table 1. Conducted between 1983 and 2021 in diverse locations (Russia, Iran, Japan, Spain, Brazil, Canada, Ireland, China, Austria, Iraq, Denmark, the USA, and Egypt), the studies enrolled participants aged 8–113 years with a wide range of conditions. These included healthy individuals, post-surgical patients, and individuals with conditions such as heart failure, hypertension, coronary heart disease, heart valve defects, cardiomyopathy, type 1 diabetes mellitus, type 2 diabetes mellitus, obesity, alcoholism, and homocystinuria.

### Quality assessment

Eighteen studies [8, 17–25, 27, 28, 30, 33–38] lacked information on allocation concealment, putting them at risk of bias. The remaining seven studies [7, 26, 29, 31, 32, 35, 39] had a low risk of bias, and none had a high risk of bias (Fig. S1, Table 3).

**Table 3:** Detailed quality assessment of the included studies using Cochrane risk of bias 2 tool.

First Author	Year	Randomization process	Intervention adherence	Missing outcome data	Outcome measurement	Selective reporting	Overall RoB
Azuma	1983	S^a^	L	L	L	L	S
Azuma	1985	S^a^	L	L	L	L	S
Fujita	1987	S^a^	L	L	L	L	S
Azuma	1992	S^a^	L	L	L	L	S
Mizushima	1996	S^a^	L	L	L	L	S
Zhang	2004	S^a^	L	L	L	L	S
Spohr	2005	L	L	L	L	L	L
Moloney	2010	L	L	L	L	L	L
Beyranva-nd	2011	S^a^	L	L	L	L	S
Arrieta	2014	S^a^	L	L	L	L	S
Hsieh	2014	S^a^	L	L	L	L	S
Rosa	2014	S^a^	L	L	L	L	S
Averin	2015	S^a^	L	L	L	L	S
Ra	2016	S^a^	L	L	L	L	S
Sun	2016	S^a^	L	L	L	L	S
Ahmadian	2017	S^a^	L	L	L	L	S
Ahmadian	2017	S^a^	L	L	L	L	S
Schwarzer	2018	L	L	L	L	L	L
Batitucci	2019	S^a^	L	L	L	L	S
Shari	2019	S^a^	L	L	L	L	S
Van Hove	2019	S^a^	L	L	L	L	S
Maleki	2020	L	L	L	L	L	L
Esmaeili	2021	L	L	L	L	L	L
Zaki	2021	L	L	L	L	L	L
Moludi	2022	L	L	L	L	L	L

*H* high risk of bias, *L* low risk of bias, *RoB* risk of bias, *S* some risk of bias.

^a^The studies did not provide allocation concealment details.

### Primary outcomes

#### Effects of taurine on SBP/DBP

**Fig. 2:**
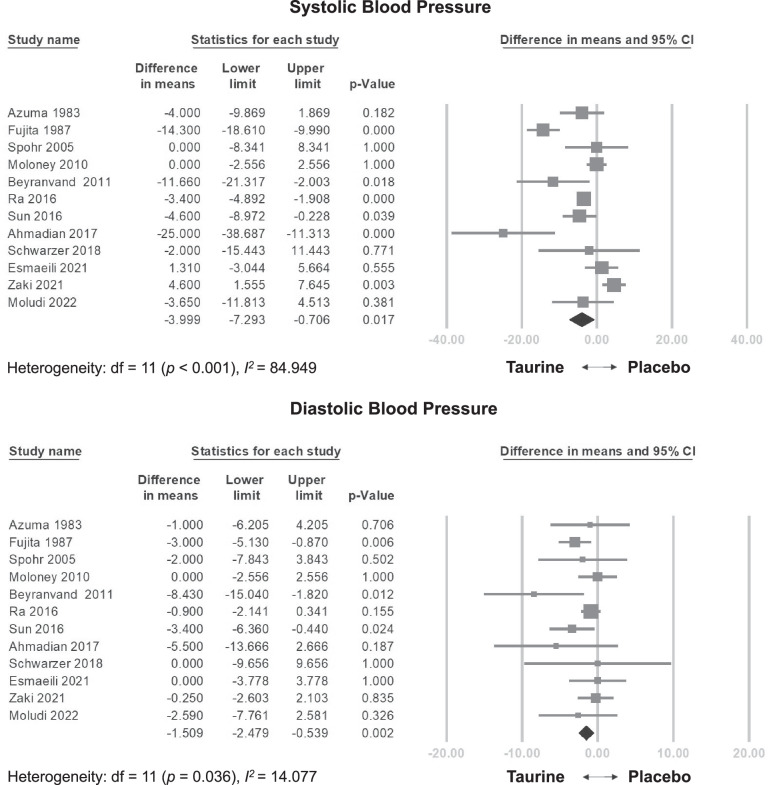
Forest plot of overall effects of taurine on systolic blood pressure (SBP) and diastolic blood pressure (DBP).

Taurine supplementation significantly reduced SBP compared to the control group (WMD = −3.999 mmHg, 95% CI = −7.293 to −0.706, *p* = 0.017, I^2^ = 84.949) (Fig. 2a). This effect remained consistent even after excluding individual studies on the sensitivity analysis (Fig. S2a). Meta-analysis regression did not reveal a statistically significant linear relationship between total dose and SBP (coefficient = −0.024 mmHg per g, *p* = 0.113) (Fig. S3a), and a significant relationship between daily dose and SBP (coefficient = −1.1258 mmHg per g/day, *p* = 0.0055) (Fig. S4a).

Taurine significantly reduced DBP levels (WMD = −1.509 mmHg, 95% CI = −2.479 to −0.539, *p* = 0.002, I^2^ = 14.077) (Fig. 2b). Similar to SBP, this DBP reduction persisted in the sensitivity analysis (Fig. S2b). Moreover, meta-regression analysis showed a significant correlation between total dose and decreased DBP (coefficient = −0.014 mmHg per g, *p* = 0.026) (Fig. S3b), and a significant relationship between daily dose and DBP (coefficient = −0.3247 mmHg per g/day, *p* = 0.0182) (Fig. S4b).

#### Effects of taurine on FBG

**Fig. 3:**
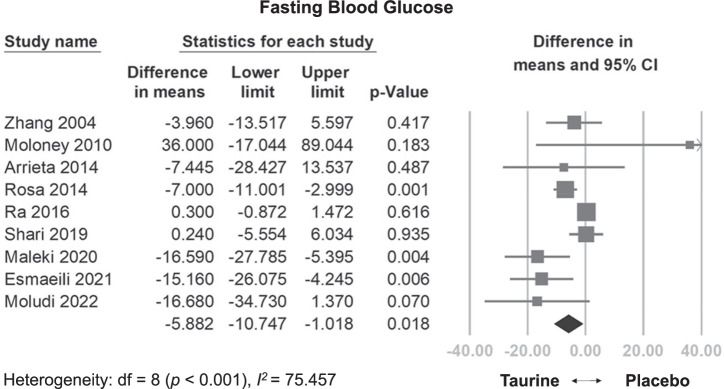
Forest plot of overall effects of taurine on fasting blood glucose (FBG).

Overall, taurine supplementation significantly reduced FBG levels compared to the control group (WMD: −5.882 mg/dL, 95% CI: −10.747 to −1.018, *p* = 0.018, I^2^ = 75.457) (Fig. 3). This effect remained consistent even after excluding individual studies in the sensitivity analysis (Fig. S5). Interestingly, meta-regression revealed a significant correlation between total dose and decreased FBG levels (coefficient = −0.495 mg/dL per g, *p* = 0.0011) (Fig. S6), but no significant relationship between daily dose and FBG (coefficient = −1.5146 mg/dL per g/day, *p* = 0.0703) (Fig. S7).

#### Effects of taurine on TG

**Fig. 4:**
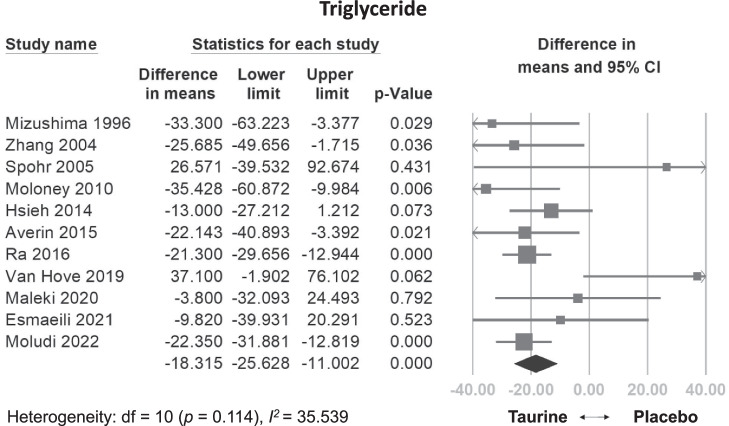
Forest plot of overall effects of taurine on triglyceride (TG).

Taurine supplementation significantly reduced TG levels compared to the control group (WMD: −18.315 mg/dL, 95% CI: −25.628 to −11.002, *p* < 0.001, I^2^ = 35.539) (Fig. 4). This effect remained consistent even after excluding individual studies in a sensitivity analysis (Fig. S8). While meta-regression did not reveal a statistically significant dose-dependent relationship between total dose and TG reduction (coefficient = −0.0522 mg/dL per g, *p* = 0.0730) (Fig. S9), it revealed a significant relationship between daily dose and TG (coefficient = −3.3600 mg/dL per g/day, *p* = 0.0062) (Fig. S10).

#### Effects of taurine on HDL-C

**Fig. 5:**
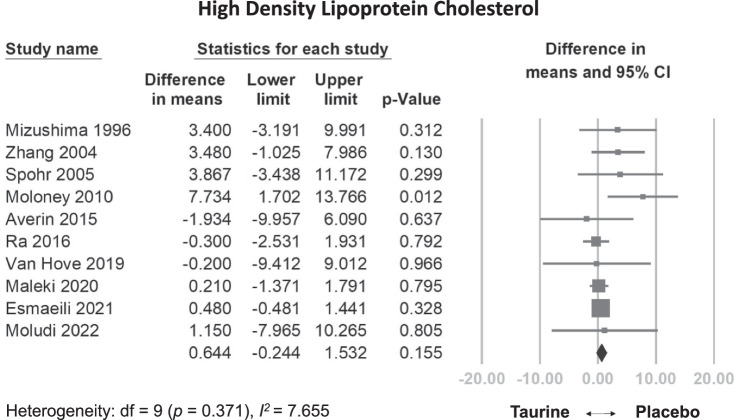
Forest plot of overall effects of taurine on high density lipoprotein-cholesterol (HDL-C).

Overall, taurine supplementation did not significantly increase HDL-C levels compared to the control group (WMD: 0.644 mg/dL, 95% CI: −0.244 to 1.532, *p* = 0.155, I^2^ = 7.655) (Fig. 5). This observation remained consistent in the sensitivity analysis (Fig. S11). Similarly, meta-regression did not show a statistically significant dose-dependent relationship between total dose and HDL-C levels (coefficient = 0.0037 mg/dL per g, *p* = 0.2729) (Fig. S12). Moreover, it didn’t reveal a significant relationship between daily dose and HDL-C (coefficient = 0.1370 mg/dL per g/day, *p* = 0.3200) (Fig. S13).

#### Publication bias

Funnel plot analysis for all investigated outcomes (SBP, DBP, FBG, TG, and HDL-C) indicated no evidence of publication bias. The distribution effect sizes were symmetric, as confirmed by Egger’s regression test, with *p* values exceeding 0.5 for all outcomes (*p* = 0.439, *p* = 0.213, *p* = 0.083, *p* = 0.166, and *p* = 0.158, respectively) (Figs. S14–S17).

### Secondary outcomes

#### Effects of taurine on body composition

Taurine supplementation did not significantly impact BW or BMI compared to the control group. The pooled effect size for BW change was minimal and non-significant (WMD: −0.642 kg, 95% CI: −1.494 to 0.209, *p* = 0.139) (Fig. S18a). Similarly, the effect size for BMI change was not statistically significant (WMD: −0.296 kg, 95% CI: −0.889 to 0.296, *p* = 0.327) (Fig. S18b). These findings were further supported by a sensitivity analysis with consistent non-significant effects of taurine on both BW and BMI (Fig. S18c, d).

#### Effects of taurine on lipid profiles

Taurine demonstrated a significant beneficial effect on lipid profiles. Compared to the control group, taurine supplementation significantly reduced both TC and LDL-C levels. The pooled effect size showed a notable decrease in TC (WMD: −8.305 mg/dL, 95% CI: −13.771 to −2.929, *p* = 0.003) (Fig. S19a), and a similar statistically significant effect was observed for LDL-C levels (WMD: −6.495 mg/dL, 95% CI: −10.912 to −2.079, *p* = 0.004) (Fig. S19b). These findings were further validated by a sensitivity analysis showing consistent significant effects of taurine on both TC and LDL-C reduction (Fig. S19c and Fig. S19d).

#### Effects of taurine on glycemic status

Taurine supplementation positively impacted several glycemic markers. Pooled effect sizes revealed significant reductions in HbA1c (WMD: −0.341%, 95% CI: −0.709 to −0.028, *p* = 0.070) (Fig. S20a), HOMA index (WMD: −0.693, 95% CI: −1.133 to −0.252, *p* = 0.002) (Fig. S20b), and fasting insulin levels (WMD: −1.521 mU/L, 95% CI: −2.591 to −0.451, *p* = 0.005) (Fig. S20c) compared to the control group. A sensitivity analysis showed a consistently non-significant effect on HbA1c reduction (Fig. S20d) but maintained consistent significant effects on both HOMA and fasting insulin (Fig. S20e, f).

#### Adverse effects

Meta-analysis of the treatment-associated adverse effect rates showed no significant differences between the taurine and control groups (odds ratio = 1.481, 95% CI = 0.843–2.604, *p* = 0.172) (Fig. S17).

## Discussion

Our meta-analysis found that taurine supplementation significantly reduced SBP, DBP, FBG, and TG levels, suggesting an improvement in risk factors associated with MetS. A dose-dependent effect is evident on SBP, DBP, FBG, and TG levels, as influenced by both dose per day and total dose. Even though not all parameters reached statistical significance, the consistent trends point to a higher physiological response to administered substance doses. However, our analysis revealed no discernible dose-dependent effect on HDL-C levels, irrespective of dose per day or total dose. Notably, taurine did not exhibit any apparent adverse effects.

Compared to the control group, taurine demonstrated a significant lowering of both SBP and DBP. The hypotensive effect may be attributed to several mechanisms, primarily through increased nitric oxide availability [15] and enhanced hydrogen sulfide production [14], ultimately leading to improved blood flow dilation [13]. Our findings also revealed a remarkable ability of taurine to effectively reduce FBG levels. This suggests a positive impact on glycemic control, potentially via various mechanisms. These include: reduced hepatic glucose production, inhibition of glucagon activity, elevated uncoupling protein 1 levels [40], enhanced insulin clearance by insulin-degrading enzymes [41], and support for the health of beta-pancreatic cells [42]. Furthermore, taurine may upregulate adiponectin mRNA expression and increase blood adiponectin levels, thereby improving insulin sensitivity [43] and contributing to overall metabolic health.

A previous meta-analysis by Guan et al. [44] found no significant difference in FBG between taurine and control groups, our results revealed a notable reduction. This discrepancy may be due to our inclusion of more studies involving individuals with diabetes, where baseline glucose levels were already elevated [19, 26, 29, 32]. This suggests that taurine’s impact might be less pronounced in those with normal blood sugar regulation.

Taurine significantly reduced TG levels. This is likely due to its ability to enhance TG removal into the bile by stimulating the production of bile acid through heightened hepatic cholesterol 7α-hydroxylase activity. Additionally, taurine increases LDL-C receptor activity, aiding in LDL-C clearance from the blood [45].

While our meta-analysis did not show a statistically significant increase in HDL-C levels, the observed trends suggest potential benefits. HDL serves as a crucial form of endogenous lipid storage by removing excess cholesterol from peripheral tissues. Hepatocytes and intestinal cells are the main internal biosynthesisers that control its levels [46]. Taurine has known effects on promoting cholesterol catabolism, particularly by increasing CYP7A1 activity and bile acid synthesis, which enhances cholesterol removal through feces. Additionally, taurine decreases the expression of ApoB-100 and ApoE, which are major receptors for LDL and VLDL, further aiding in cholesterol clearance [47]. It’s likely that taurine efficiently removes cholesterol, displacing the need for extra HDL clearance. Furthermore, although taurine has been shown to raise serum HDL levels in rats raised on a high-cholesterol diet [47], human trials usually do not include purposefully high-fat diets, and the majority of participants are not obese. Therefore, in contrast to animal research, the effects of taurine on HDL levels in human trials may be less apparent. It is noteworthy that these trends towards elevated HDL-C may have a noteworthy effect on lowering the atherosclerosis index, even in the absence of statistically significant results [48].

Beyond FBG, taurine demonstrates effectiveness in both glycemic control and lipid profiles, with significant reductions observed in TC, LDL-C, fasting insulin, and HOMA levels. Although our analysis revealed only marginal benefits on HbA1c (WMD -0.341 [95% CI: −0.709, −0.28], *p* = 0.07), this may be attributed to the limited duration and intensity of protocols in the included studies (dosages ranging from 3 to 168 g with tracking periods of eight weeks to three months). Conversely, a meta-analysis conducted by Tao et al. exclusively on patients with diabetes reported significant reductions in HbA1c levels (WMD −0.41 [95% CI: −0.74, −0.09], *p* = 0.01) [5]. All four of the included trials [26, 29, 32, 36] assessing HbA1c as an endpoint involved individuals with type 2 diabetes, indicating a lack of evidence regarding HbA1c level changes in participants with diabetes. The lack of studies conducted on populations without diabetes could be because these changes are unlikely in this population. Future research may delve deeper into this topic. Since HbA1c not only offers a trustworthy indicator of chronic hyperglycemia but also strongly correlates with the risk of long-term diabetes complications [49], more research is required to fully understand its impact on long-term glycemic control in larger populations.

Taurine did not appear to significantly affect BW or BMI. In the only trial demonstrating a decrease in body mass, Shari et al. [36] administered 1 g taurine daily for 3 months to participants with type 2 diabetes. However, findings from other trials consistently showed negligible effects on body mass within their respective protocols. This suggests that factors like hypocaloric diet and exercise likely have a greater impact on body weight in this context [8].

Taurine is classified as “generally considered as safe” by the United States Food and Drug Administration [50]. In line with this, our study did not reveal any significant differences in treatment-related adverse effects between the taurine and control groups. All reported events were mild and transient, involving primarily gastrointestinal issues, headaches, and fatigue. Notably, no instances of moderate or severe events were associated with taurine supplementation [19, 35, 38].

While previous studies have examined similar endpoints [5, 44], ours is the first to conduct a meta-regression analysis on dose-response relationships, highlighting the effectiveness of taurine in mitigating MetS risk factors in the general population. However, some limitations warrant further consideration.

The observed heterogeneity has the potential to attenuate the true effect of taurine, introducing variability across different populations that may result in diminished statistical significance for parameters such as TG and HDL-C. Several factors contribute to this heightened heterogeneity. Firstly, there is variation in the selection of participants across studies, where individuals with diabetes and obesity, although at high risk for metabolic diseases, may not necessarily be diagnosed with specific disorders. Secondly, inconsistencies in study protocols play a role, with some participants adhering to their regular diet and activity levels [8], while others follow calorie-modified plans [32], potentially influencing results and increasing heterogeneity. Thirdly, our included trials suggest that taurine is more effective in moderating metabolism among studies that exclusively recruit patients with obesity or diabetes, as their TG levels are inherently higher and more prone to decrease compared to those with cardiovascular disease or no definite metabolic disorder.

When examining the impact of taurine on lipid profiles in patients with cardiovascular disease, confounding factors such as concurrent medication use must be considered. Given that a large number of the included studies focus on patients with heart failure, it is important to remember that medications like diuretics, angiotensin-converting enzyme (ACE), and vasodilators, which are commonly prescribed for cardiovascular conditions, can affect lipid levels. Long-term use of ACE inhibitors like enalapril has shown to significantly reduce TC, TG, and Very Low-density lipoprotein (VLDL) in patients [51]. Thiazide-type diuretics, often used for hypertension, may increase TC and VLDL levels in the short term, while HDL-C levels remain unchanged [52].

The authors of some included trials [26, 29, 32] were more aware of the impact of lipid level resulting from concurrent medication use, and specifically instructed participants to maintain their standard therapeutic regimen, diet, and lifestyle, with careful monitoring and controlled for. To better understand taurine’s effects on lipid profiles, future clinical trials should carefully consider and possibly exclude patients using medications known to significantly impact lipid levels.

Direct data on waist circumference, another key MetS criterion, were unavailable for any of the included trials. Despite this, we incorporated BMI as a secondary outcome, which could indirectly reflect the potential impact of taurine on waist circumference due to their strong correlation [53].

Another critical issue is that the majority of included studies on taurine’s effects typically have short durations, lasting no more than two months, with only a few extending up to a year at most. This limited timeframe emphasizes the necessity for longer-term studies to validate taurine’s efficacy thoroughly. Future studies should conduct more future studies to ascertain the duration of taurine’s effects after its cessation. Such extended investigations are crucial to explore its potential incorporation into novel clinical guidelines for the management of MetS and related conditions.

## Conclusion

In conclusion, our meta-analysis of RCTs highlights taurine supplementation’s significant potential in mitigating key MetS risk factors, including reductions in SBP, DBP, FBG, and TG levels. This underscores its potential as a complementary therapeutic agent for MetS management, offering a multifaceted approach to glycemic control and cardiovascular health. Future clinical trials should focus on determining optimal taurine dosage and treatment duration, especially in MetS-susceptible populations. Addressing limitations in existing trials, such as variations in dosage, trial duration, sample size, disease severity, and participant characteristics, is crucial for generating robust evidence. As taurine is an inexpensive and easily obtainable supplement, further research can fill knowledge gaps, supporting clinical guidelines on its use as a nutraceutical for MetS prevention and management. Integrating taurine supplementation with established pharmacological interventions may enhance treatment outcomes and overall cardiovascular health in MetS patients.

### Supplementary information

Supplemental material

## Data Availability

All data included in this study are shown in this article or supplementary information, any further request is available by contacting the corresponding authors.
