# Supplementary material for: Taurine reduces the risk for metabolic syndrome: a systematic review and meta-analysis of randomized controlled trials
Source: Nutr Diabetes. 2024 May 16;14:29. doi: 10.1038/s41387-024-00289-z (PMC11099170; doi:10.1038/s41387-024-00289-z)
Supplement: Supplementary file 1 — Supplemental material [file 41387_2024_289_MOESM1_ESM.docx]

**Supplemental Material**

**Table S1**. PRISMA Checklist

| **Section and Topic** | **#** | **Checklist item** | **Location** |
| --- | --- | --- | --- |
| **TITLE** | | |  |
| Title | 1 | Identify the report as a systematic review. | Title |
| **ABSTRACT** | | |  |
| Abstract | 2 | See the PRISMA 2020 for Abstracts checklist. | Abstract |
| **INTRODUCTION** | | |  |
| Rationale | 3 | Describe the rationale for the review in the context of existing knowledge. | Introduction |
| Objectives | 4 | Provide an explicit statement of the objective(s) or question(s) the review addresses. | Introduction |
| **METHODS** | | |  |
| Eligibility criteria | 5 | Specify the inclusion and exclusion criteria for the review and how studies were grouped for the syntheses. | Methods |
| Information sources | 6 | Specify all databases, registers, websites, organisations, reference lists and other sources searched or consulted to identify studies. Specify the date when each source was last searched or consulted. | Methods |
| Search strategy | 7 | Present the full search strategies for all databases, registers and websites, including any filters and limits used. | Methods, Table S2 |
| Selection process | 8 | Specify the methods used to decide whether a study met the inclusion criteria of the review, including how many reviewers screened each record and each report retrieved, whether they worked independently, and if applicable, details of automation tools used in the process. | Methods |
| Data collection process | 9 | Specify the methods used to collect data from reports, including how many reviewers collected data from each report, whether they worked independently, any processes for obtaining or confirming data from study investigators, and if applicable, details of automation tools used in the process. | Methods |
| Data items | 10a | List and define all outcomes for which data were sought. Specify whether all results that were compatible with each outcome domain in each study were sought (e.g., for all measures, time points, analyses), and if not, the methods used to decide which results to collect. | Methods |
|  | 10b | List and define all other variables for which data were sought (e.g., participant and intervention characteristics, funding sources). Describe any assumptions made about any missing or unclear information. | Methods, Table 1-2 |
| Study risk of bias assessment | 11 | Specify the methods used to assess risk of bias in the included studies, including details of the tool(s) used, how many reviewers assessed each study and whether they worked independently, and if applicable, details of automation tools used in the process. | Methods |
| Effect measures | 12 | Specify for each outcome the effect measure(s) (e.g., risk ratio, mean difference) used in the synthesis or presentation of results. | Methods |
| Synthesis methods | 13a | Describe the processes used to decide which studies were eligible for each synthesis (e.g., tabulating the study intervention characteristics and comparing against the planned groups for each synthesis (item #5)). | Methods, Figure 1,  Table 1-2, Table S3 |
|  | 13b | Describe any methods required to prepare the data for presentation or synthesis, such as handling of missing summary statistics, or data conversions. | Methods |
|  | 13c | Describe any methods used to tabulate or visually display results of individual studies and syntheses. | Methods |
|  | 13d | Describe any methods used to synthesize results and provide a rationale for the choice(s). If meta-analysis was performed, describe the model(s), method(s) to identify the presence and extent of statistical heterogeneity, and software package(s) used. | Methods |
|  | 13e | Describe any methods used to explore possible causes of heterogeneity among study results (e.g., subgroup analysis, meta-regression). | Methods |
|  | 13f | Describe any sensitivity analyses conducted to assess robustness of the synthesized results. | Methods |
| Reporting bias assessment | 14 | Describe any methods used to assess risk of bias due to missing results in a synthesis (arising from reporting biases). | Methods, Figure S1,  Table 3 |
| Certainty assessment | 15 | Describe any methods used to assess certainty (or confidence) in the body of evidence for an outcome. | Methods |
| **RESULTS** | | |  |
| Study selection | 16a | Describe the results of the search and selection process, from the number of records identified in the search to the number of studies included in the review, ideally using a flow diagram. | Results, Figure 1,  Table S2-S3 |
|  | 16b | Cite studies that might appear to meet the inclusion criteria, but which were excluded, and explain why they were excluded. | Results, Table S3 |
| Study characteristics | 17 | Cite each included study and present its characteristics. | Results, Table 1-2 |
| Risk of bias | 18 | Present assessments of risk of bias for each included study. | Table 3, Figure S1 |
| Results of individual studies | 19 | For all outcomes, present, for each study: (a) summary statistics for each group (where appropriate) and (b) an effect estimates and its precision (e.g., confidence/credible interval), ideally using structured tables or plots. | Figure 2-5, Figure S2-S17 |
| Results of syntheses | 20a | For each synthesis, briefly summarise the characteristics and risk of bias among contributing studies. | Results, Table 3 |
|  | 20b | Present results of all statistical syntheses conducted. If meta-analysis was done, present for each the summary estimate and its precision (e.g., confidence/credible interval) and measures of statistical heterogeneity. If comparing groups, describe the direction of the effect. | Results, Figure 2-5, Figure S2-S17 |
|  | 20c | Present results of all investigations of possible causes of heterogeneity among study results. | Results, Figure 2-5, Figure S2-S17 |
|  | 20d | Present results of all sensitivity analyses conducted to assess the robustness of the synthesized results. | Results, Figure S2, S4, S6, S8, S14-S16 |
| Reporting biases | 21 | Present assessments of risk of bias due to missing results (arising from reporting biases) for each synthesis assessed. | Table 3, Figure S1 |
| Certainty of evidence | 22 | Present assessments of certainty (or confidence) in the body of evidence for each outcome assessed. | Figure 2-5, Figure S2-S17 |
| **DISCUSSION** | | |  |
| Discussion | 23a | Provide a general interpretation of the results in the context of other evidence. | Discussion |
|  | 23b | Discuss any limitations of the evidence included in the review. | Discussion |
|  | 23c | Discuss any limitations of the review processes used. | Discussion |
|  | 23d | Discuss implications of the results for practice, policy, and future research. | Discussion |
| **OTHER INFORMATION** | | |  |
| Registration and protocol | 24a | Provide registration information for the review, including register name and registration number, or state that the review was not registered. | Methods |
|  | 24b | Indicate where the review protocol can be accessed, or state that a protocol was not prepared. | Methods, Table S2-S3 |
|  | 24c | Describe and explain any amendments to information provided at registration or in the protocol. | Methods, Table S2-S3 |
| Support | 25 | Describe sources of financial or non-financial support for the review, and the role of the funders or sponsors in the review. | Funding |
| Competing interests | 26 | Declare any competing interests of review authors. | Conflicts of Interest |
| Availability of data, code and other materials | 27 | Report which of the following are publicly available and where they can be found: template data collection forms; data extracted from included studies; data used for all analyses; analytic code; any other materials used in the review. | Results, Table S2-S3 |

**Table S2.** Keywords and search results in different databases

| Database | Keyword | Filter | Date | Results |
| --- | --- | --- | --- | --- |
| PubMed | ('taurine' OR 'taufon') AND ('metabolic syndrome X' OR 'diabetes mellitus' OR 'obesity' OR 'hypertension' OR 'dyslipidemia' OR 'hyperglycemia') | NA | December 1st, 2023. | 1387 |
| Embase | ('taurine' OR 'taufon') AND ('metabolic syndrome X' OR 'diabetes mellitus' OR 'obesity' OR 'hypertension' OR 'dyslipidemia' OR 'hyperglycemia') [randomized controlled trial]/lim | Randomized controlled trial | December 1st, 2023. | 59 |
| Cochrane CENTRAL | ('taurine' OR 'taufon') AND ('metabolic syndrome X' OR 'diabetes mellitus' OR 'obesity' OR 'hypertension' OR 'dyslipidemia' OR 'hyperglycemia') | Trials | December 1st, 2023. | 90 |
| Web of Science | ('taurine' OR 'taufon') AND ('metabolic syndrome X' OR 'diabetes mellitus' OR 'obesity' OR 'hypertension' OR 'dyslipidemia' OR 'hyperglycemia') | NA | December 1st, 2023. | 981 |
| ClinicalTrials.gov | ('taurine' OR 'taufon') AND ('metabolic syndrome X' OR 'diabetes mellitus' OR 'obesity' OR 'hypertension' OR 'dyslipidemia' OR 'hyperglycemia') | NA | December 1st, 2023. | 17 |

NA: not applied

**Table S3.** Excluded studies and reasons

| **Citations** | **Reasons** |
| --- | --- |
| Montanini, R., & Gasco, P. (1974). [Taurine in the treatment of diffuse cerebral arteriopathies. Clinical and electroencephalographic observations and psychological tests]. Clin Ter, 71(5), 427-436. (La taurina nel trattamento delle arteriopatie cerebrali diffuse. Osservazioni cliniche, elettroencefalografiche, testali.) | Not a randomized trial |
| Fennessy, F. M., Moneley, D. S., Wang, J. H., Kelly, C. J., & Bouchier-Hayes, D. J. (2003). Taurine and vitamin C modify monocyte and endothelial dysfunction in young smokers. Circulation, 107(3), 410-415. https://doi.org/10.1161/01.Cir.0000046447.72402.47 | Not a randomized trial |
| Ahn, C. S. (2009). Effect of taurine supplementation on plasma homocysteine levels of the middle-aged Korean women. Adv Exp Med Biol, 643, 415-422. https://doi.org/10.1007/978-0-387-75681-3_43 | Not a randomized trial |
| Montanini, R., Zibetti, A., & Gasco, P. (1971). [Preliminary observations on the use of taurine in the treatment of cerebrovascular diseases]. Clin Ter, 59(4), 321-329. (Osservazioni preliminari sull'impiego della taurina nel trattamento delle vasculopatie cerebrali.) | Not a randomized trial |
| Yang, Y. Z., Chen, R. Z., & Zhang, J. N. (2001). [Observation on collaborative treatment of dilated cardiomyopathy]. Zhongguo Zhong Xi Yi Jie He Za Zhi, 21(4), 254-256. | Herbal treatment with undocumented active compounds |
| Liu, D., Lu, Z., Sun, Q., Wang, B., & Zhu, Z. (2018). Taurine supplementation improves vascular function in prehypertension: a randomized, double-blind, placebo-controlled study [Journal article; Conference proceeding]. Journal of Hypertension, 36, e145. https://doi.org/10.1097/01.hjh.0000548587.26916.dd | Poster abstract only, no available data of pre- and post-intervention endpoint. |
| Basrai, M., Schweinlin, A., Menzel, J., Mielke, H., Weikert, C., Dusemund, B., Putze, K., Watzl, B., Lampen, A., & Bischoff, S. C. (2019). Energy Drinks Induce Acute Cardiovascular and Metabolic Changes Pointing to Potential Risks for Young Adults: A Randomized Controlled Trial. Journal of Nutrition, 149(3), 441-450. https://doi.org/10.1093/jn/nxy303 | Follow up period too short to show results on metabolic syndrome |
| Adamchik, A. S., Kryuchkova, I. V., Ruban, G. M., & Blagodyreva, Y. A. (2010). New potential of pharmaceutical therapy in diastolic chronic heart failure treatment. Russian Journal of Cardiology(4), 40-43. <Go to ISI>://WOS:000282849300008 | Irrelevant outcomes |
| Gordeev, I. G., Pokrovskaya, E. M., & Luchinkina, E. E. (2012). Taurine effects on the occurrence of cardiac arrhythmias and QT interval dispersion in patients with post-infarction cardiosclerosis and chronic heart failure: a comparative randomised study. Cardiovascular Therapy and Prevention, 11(1), 63-68. <https://doi.org/10.15829/1728-8800-2012-1-63-68> | Irrelevant outcomes |
| Franconi, F., Bennardini, F., Mattana, A., Miceli, M., Ciuti, M., Mian, M., Gironi, A., Anichini, R., & Seghieri, G. (1995). Plasma and platelet taurine are reduced in subjects with insulin-dependent diabetes mellitus: Effects of taurine supplementation. American Journal of Clinical Nutrition, 61(5), 1115-1119. <https://doi.org/10.1093/ajcn/61.5.1115> | Irrelevant outcomes |
| Jeejeebhoy, F., Keith, M., Freeman, M., Barr, A., McCall, M., Kurian, R., Mazer, D., & Errett, L. (2002). Nutritional supplementation with MyoVive repletes essential cardiac myocyte nutrients and reduces left ventricular size in patients with left ventricular dysfunction. American Heart Journal, 143(6), 1092-1100. https://doi.org/10.1067/mhj.2002.121927 | Irrelevant outcomes |
| Roshan, V. D., Khalafi, M. K., & Choobineh, S. (2011). Effects of taurine supplementation on response of the cardiac injury biomarkers to bruce diagnostic protocol in patients with heart failure [Journal article]. Koomesh, 13(1), 73‐82. https://www.cochranelibrary.com/central/doi/10.1002/central/CN-00893990/full | Irrelevant outcomes |
| Singh, R. B., Kartikey, K., Charu, A. S., Niaz, M. A., & Schaffer, S. (2003). Effect of taurine and coenzyme Q10 in patients with acute myocardial infarction. In J. B. Lombardini, S. W. Schaffer, & J. Azuma (Eds.), Taurine 5: Beginning the 21st Century (Vol. 526, pp. 41-48). <Go to ISI>://WOS:000184737600005 | Irrelevant outcomes |

**Figure S1. Summary of quality assessment of studies included in the meta-analysis using Cochrane risk of bias 2 tool**

**
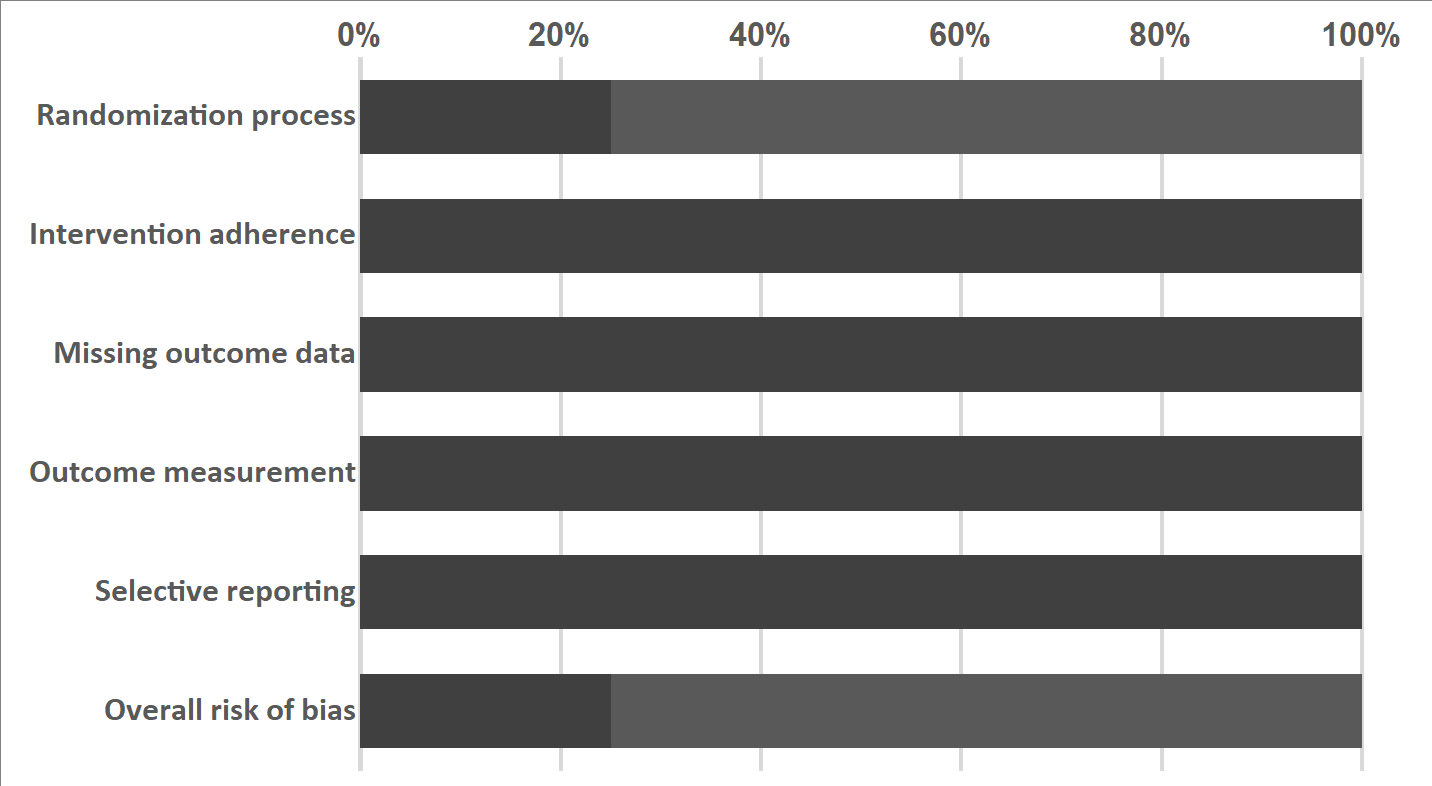
**

**Figure S2.** Results of sensitivity analysis using the one-study removal method to assess the impact of taurine on the overall effect size for (a) systolic blood pressure (SBP) (b) diastolic blood pressure (DBP)


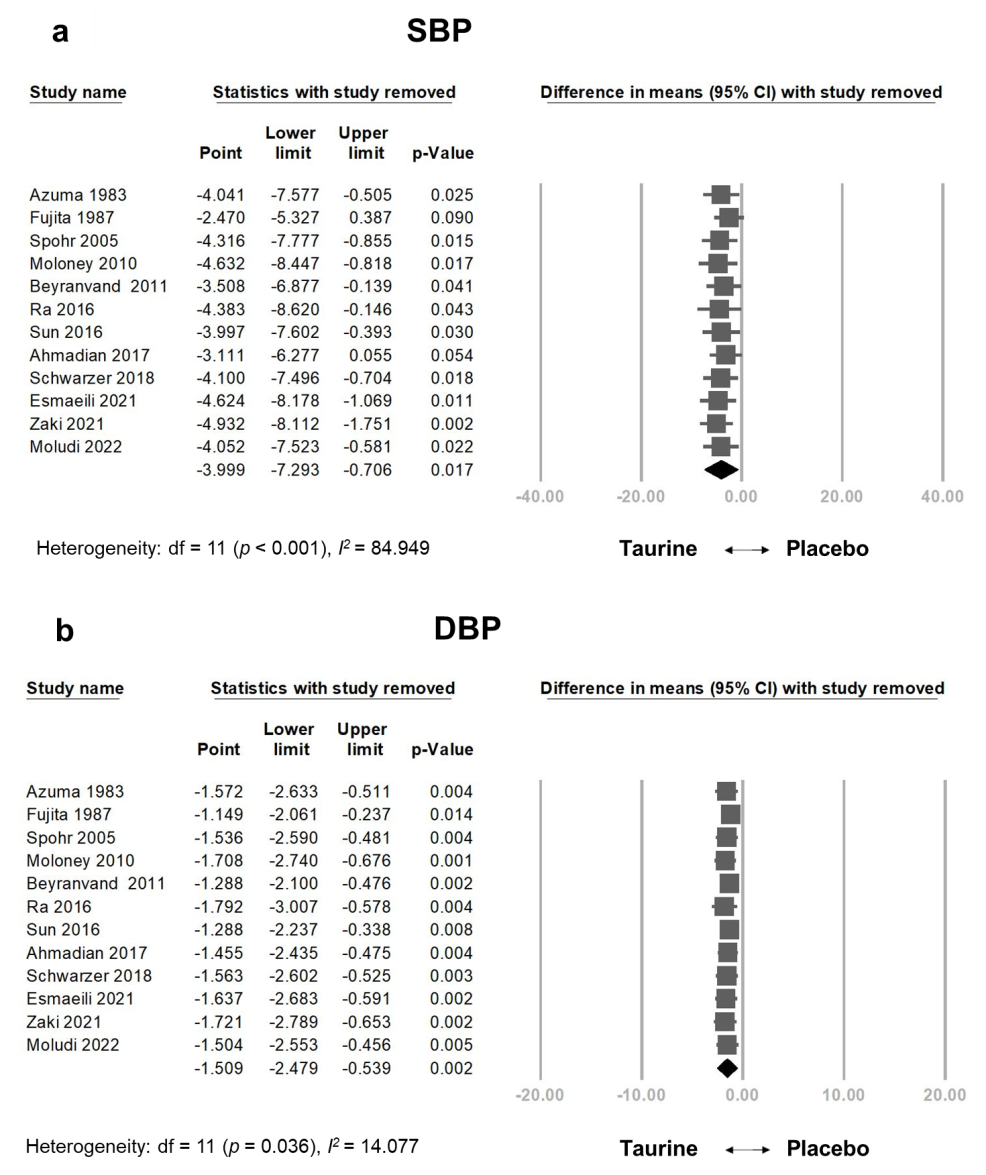


**Figure S3.** Meta-regression analysis showing the relationship between the total taurine dose throughout the treatment periods and (a) systolic blood pressure (SBP) (b) diastolic blood pressure (DBP)

**
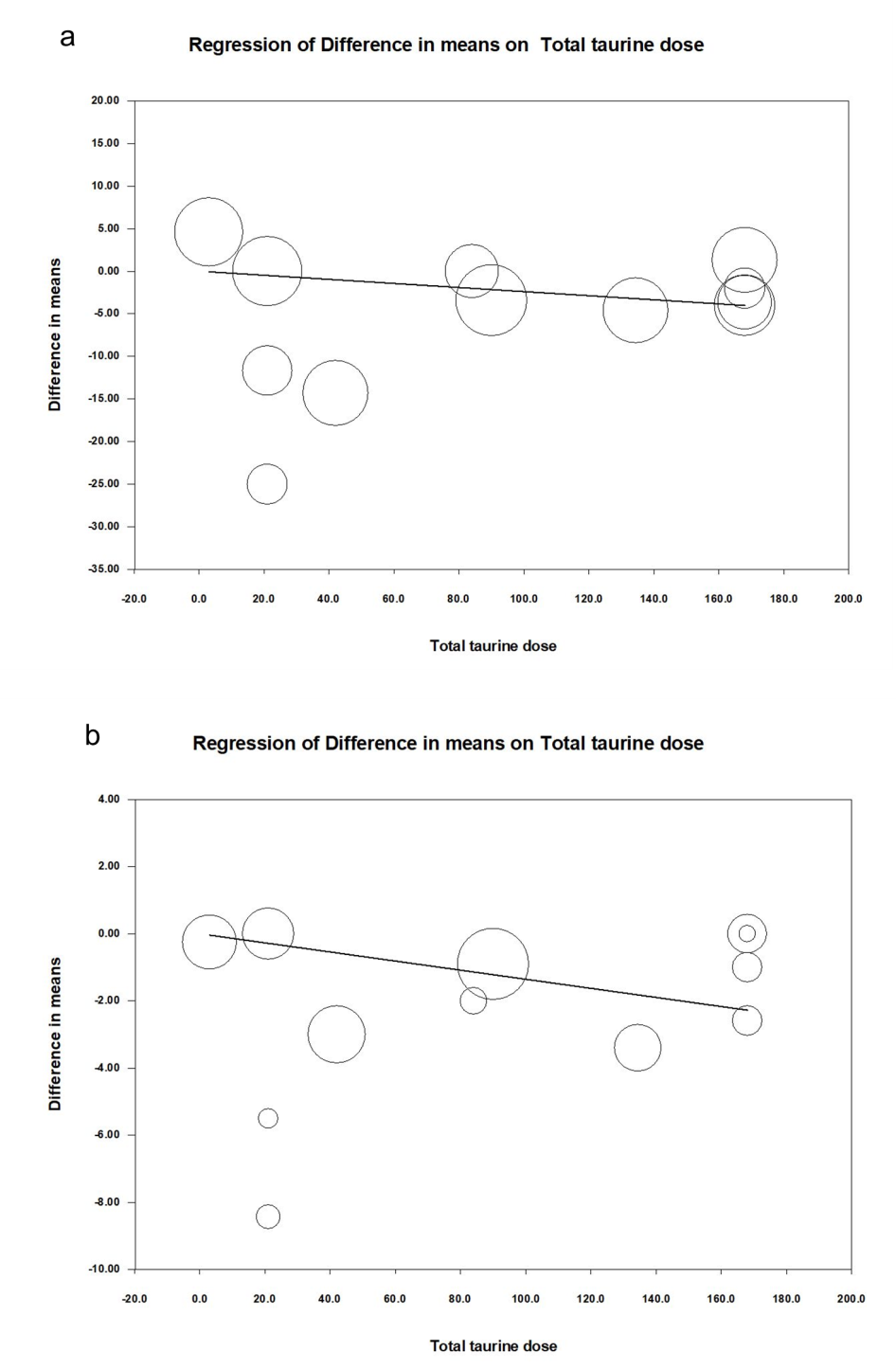
**

**Figure S4.** Meta-regression analysis showing the relationship between the daily dose of taurine and (a) systolic blood pressure (SBP) (b) diastolic blood pressure (DBP)

**
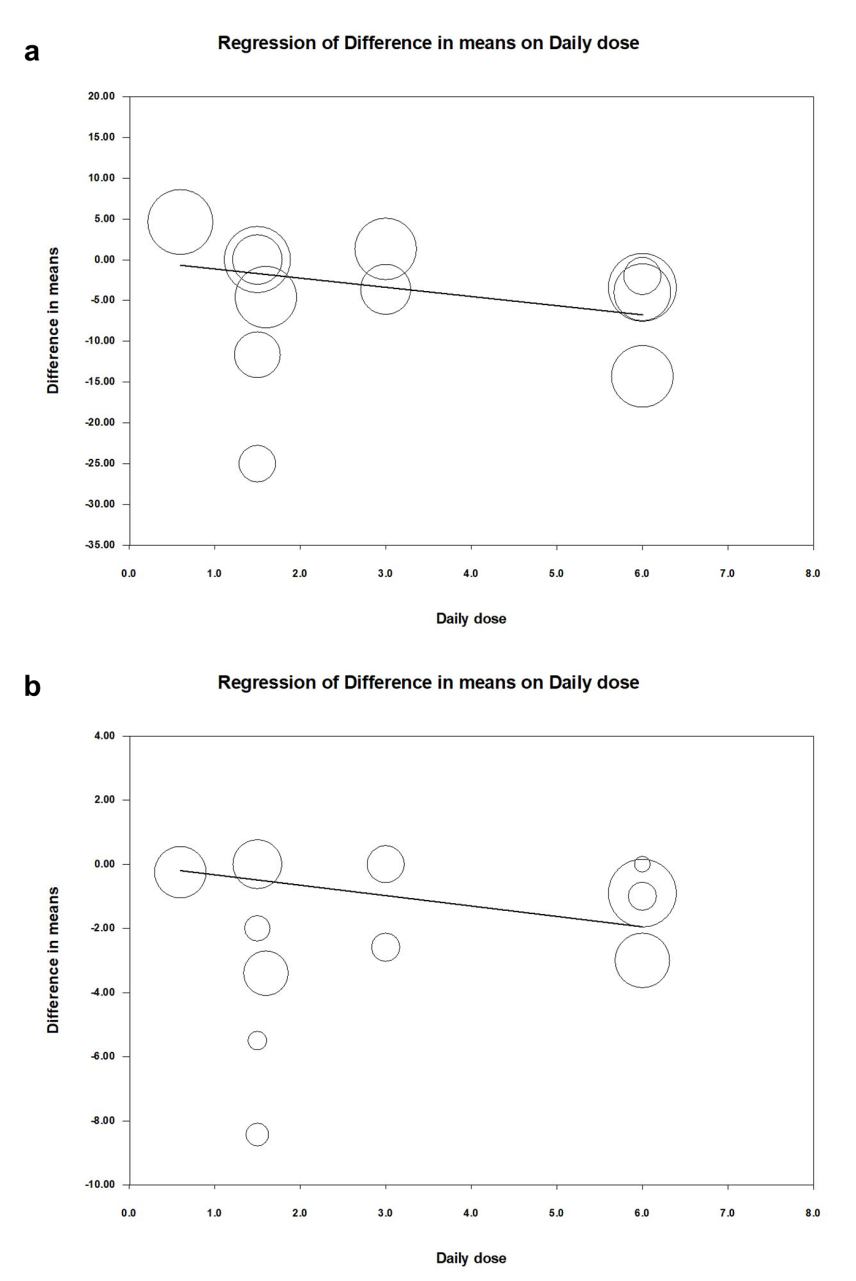
**

**Figure S5.** Results of sensitivity analysis using the one-study removal method to assess the impact of taurine on the overall effect size for fasting blood glucose (FBG)

**
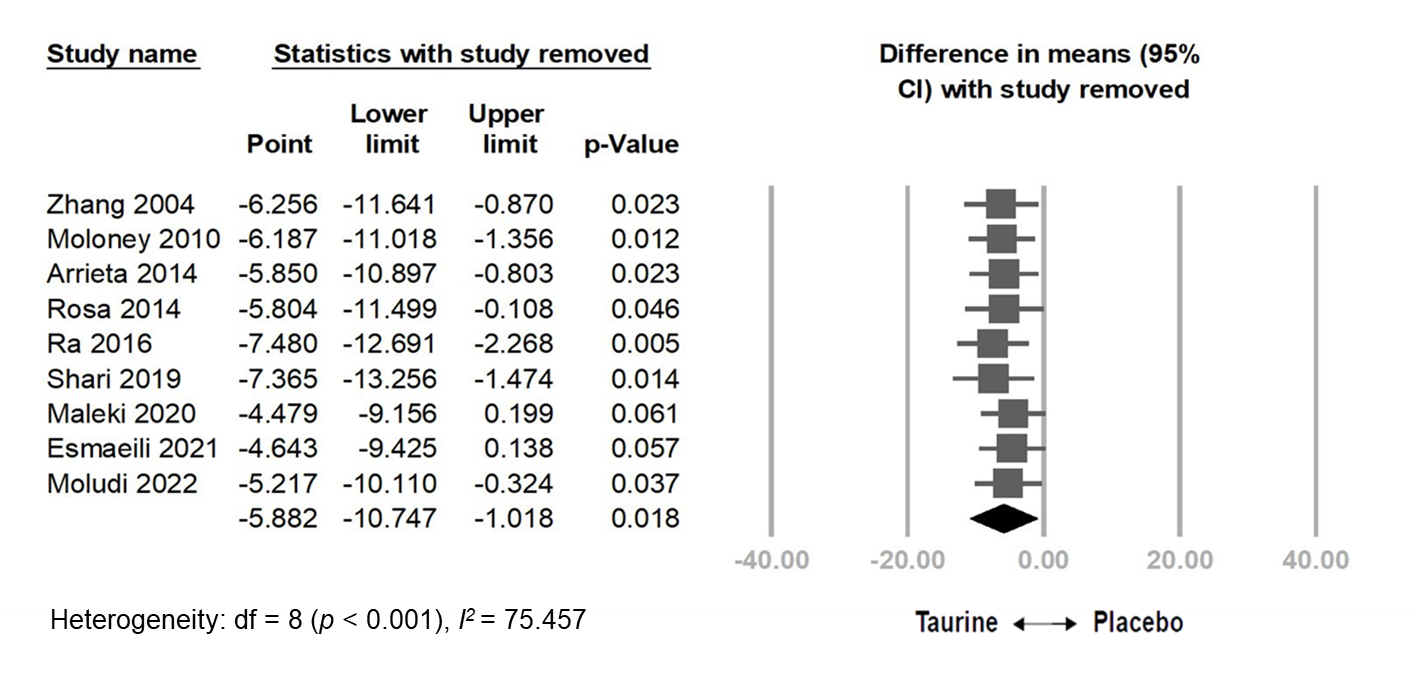
**

**Figure S6.** Meta-regression analysis showing the relationship between the total taurine dose throughout the treatment periods and fasting blood glucose (FBG)


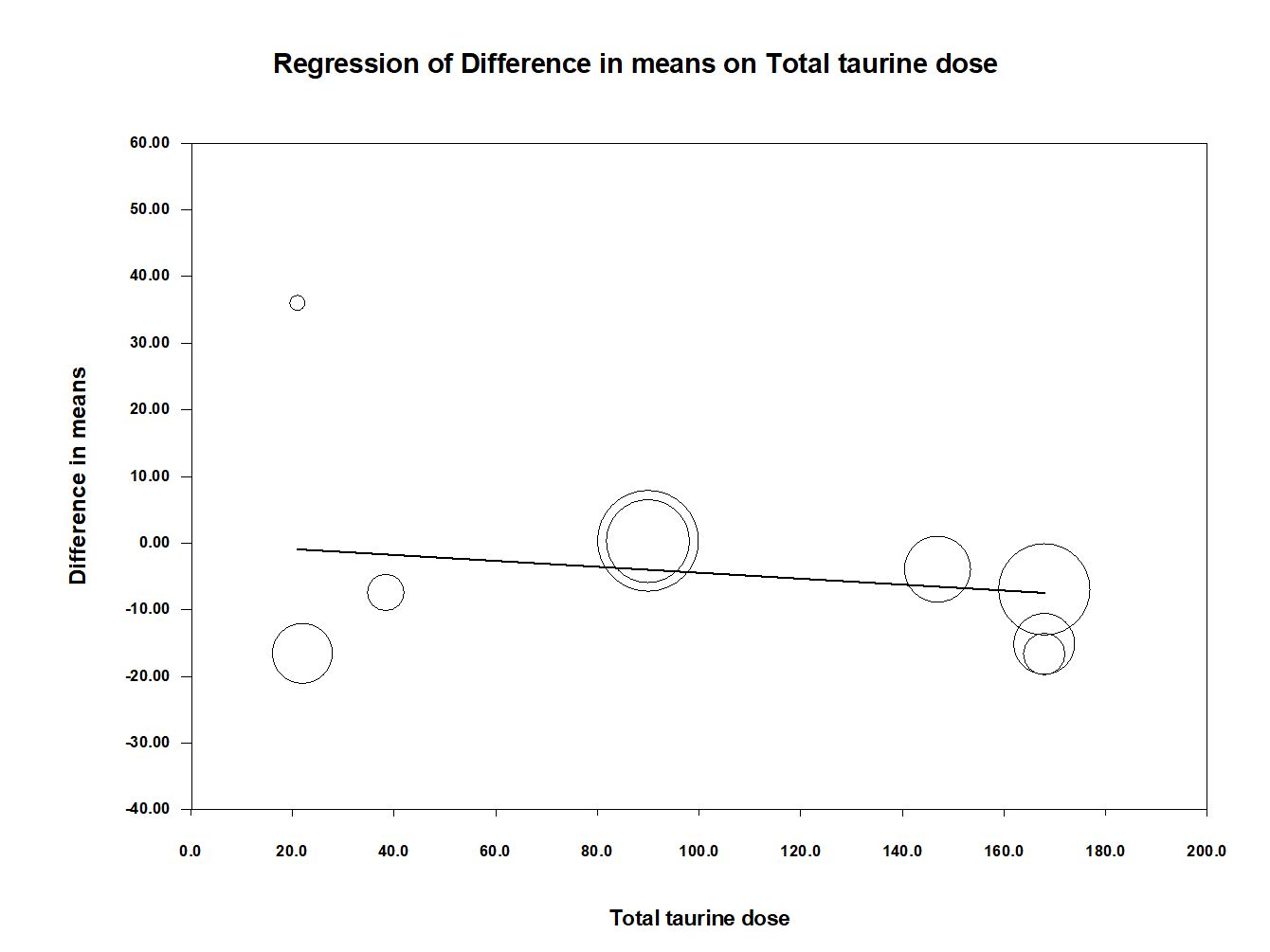


**Figure S7.** Meta-regression analysis showing the relationship between the daily dose of taurine and fasting blood glucose (FBG)


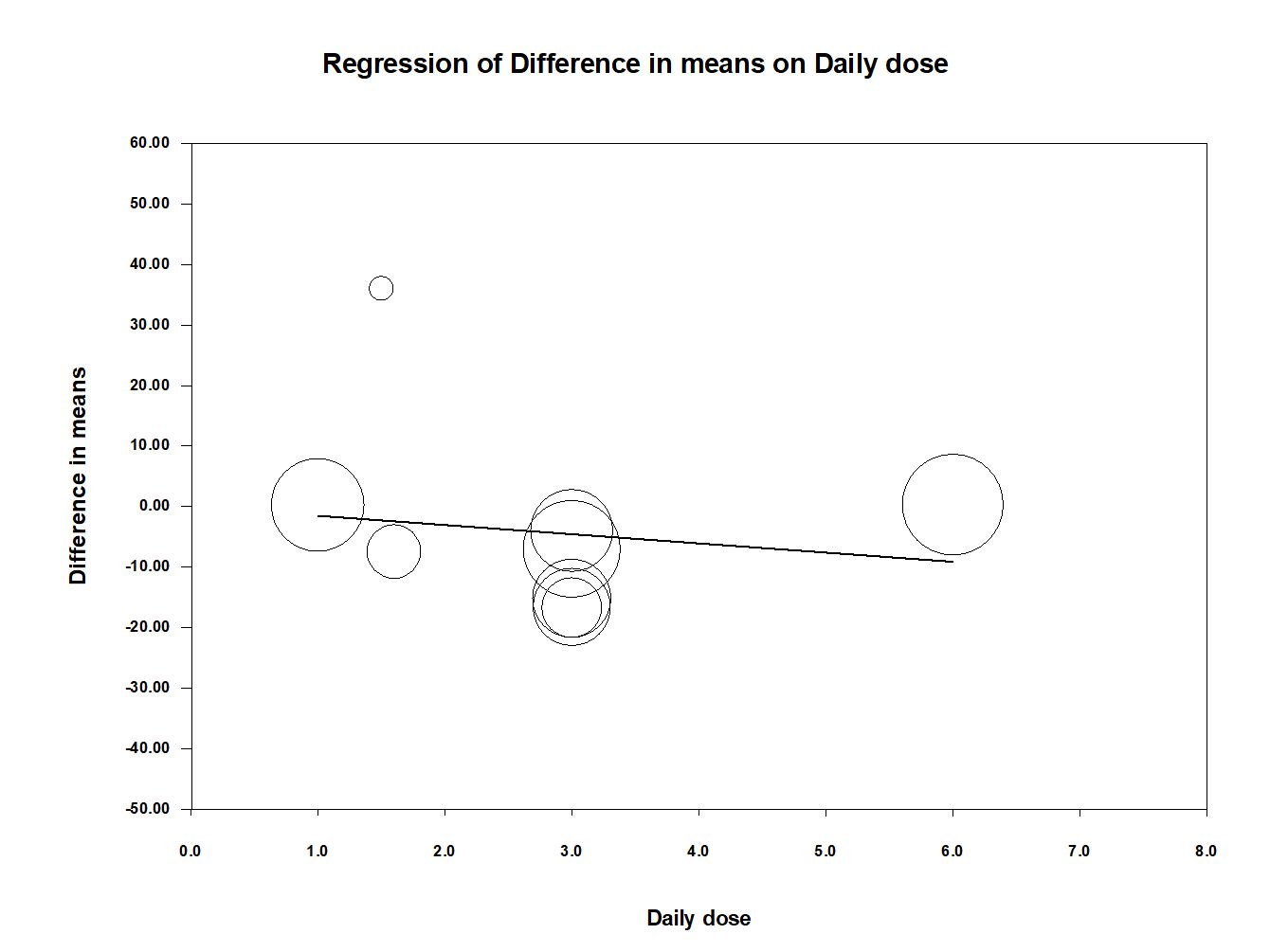


**Figure S8.** Results of sensitivity analysis using the one-study removal method to assess the impact of taurine on the overall effect size for triglyceride (TG)

**
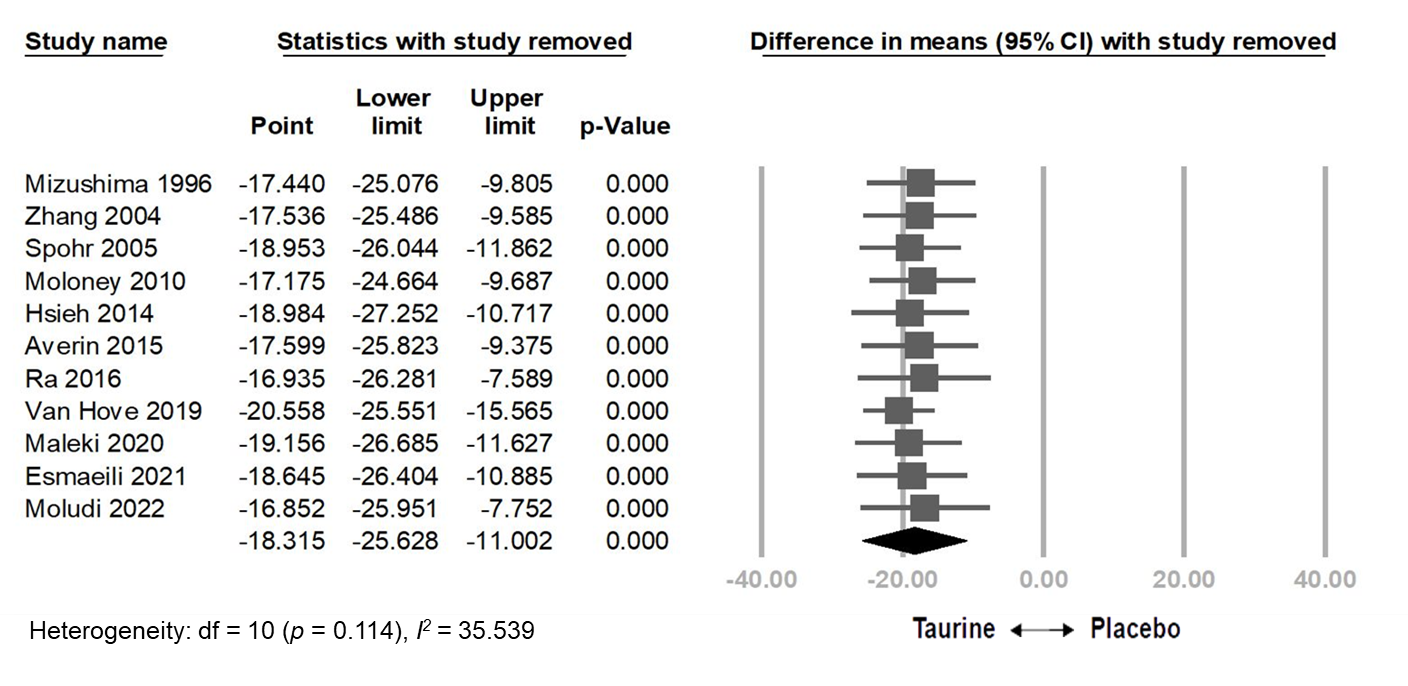
**

**Figure S9.** Meta-regression analysis showing the relationship between the total taurine dose throughout the treatment periods and triglyceride reduction


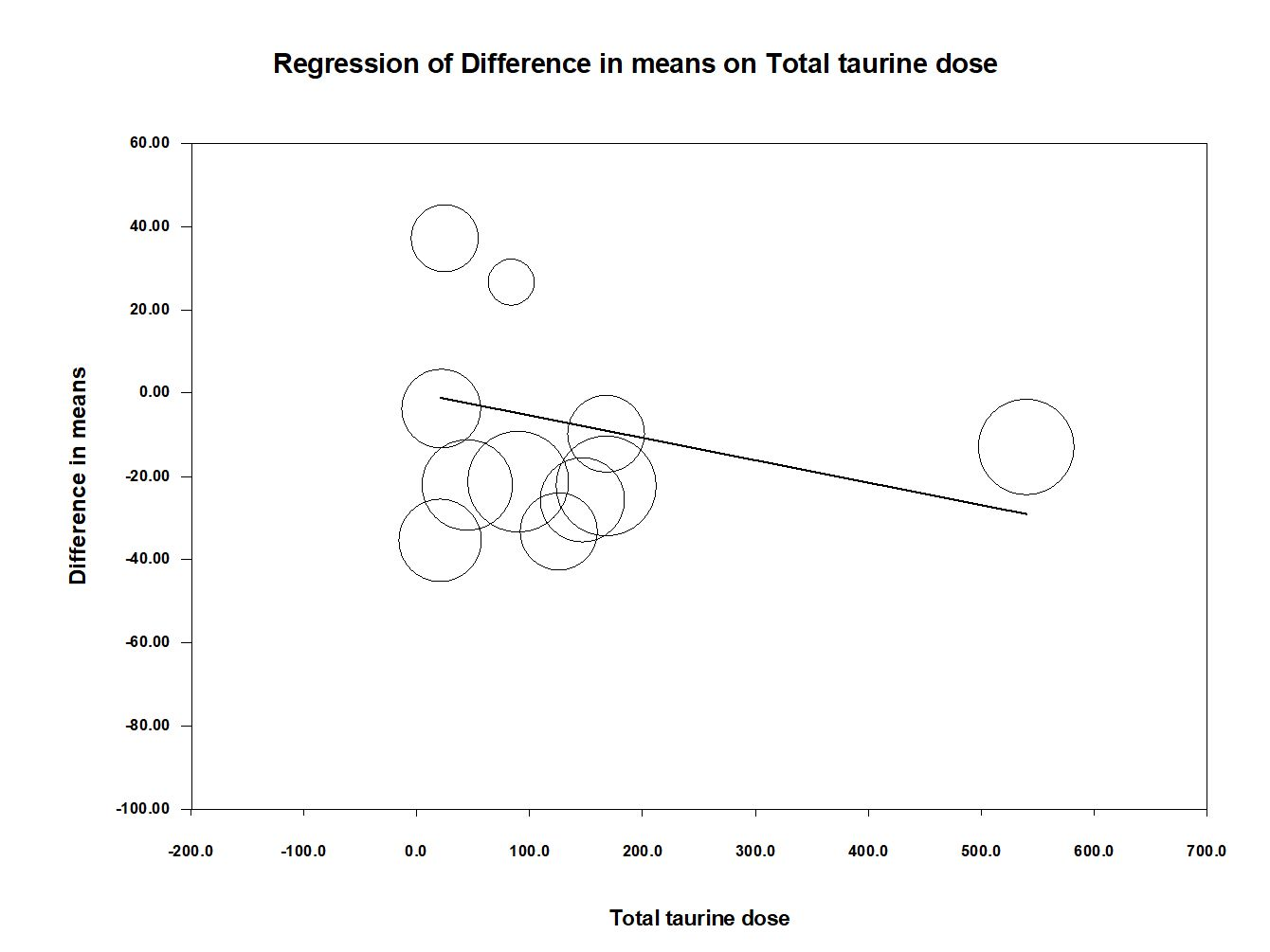


**Figure S10.** Meta-regression analysis showing the relationship between the daily dose of taurine and triglyceride reduction


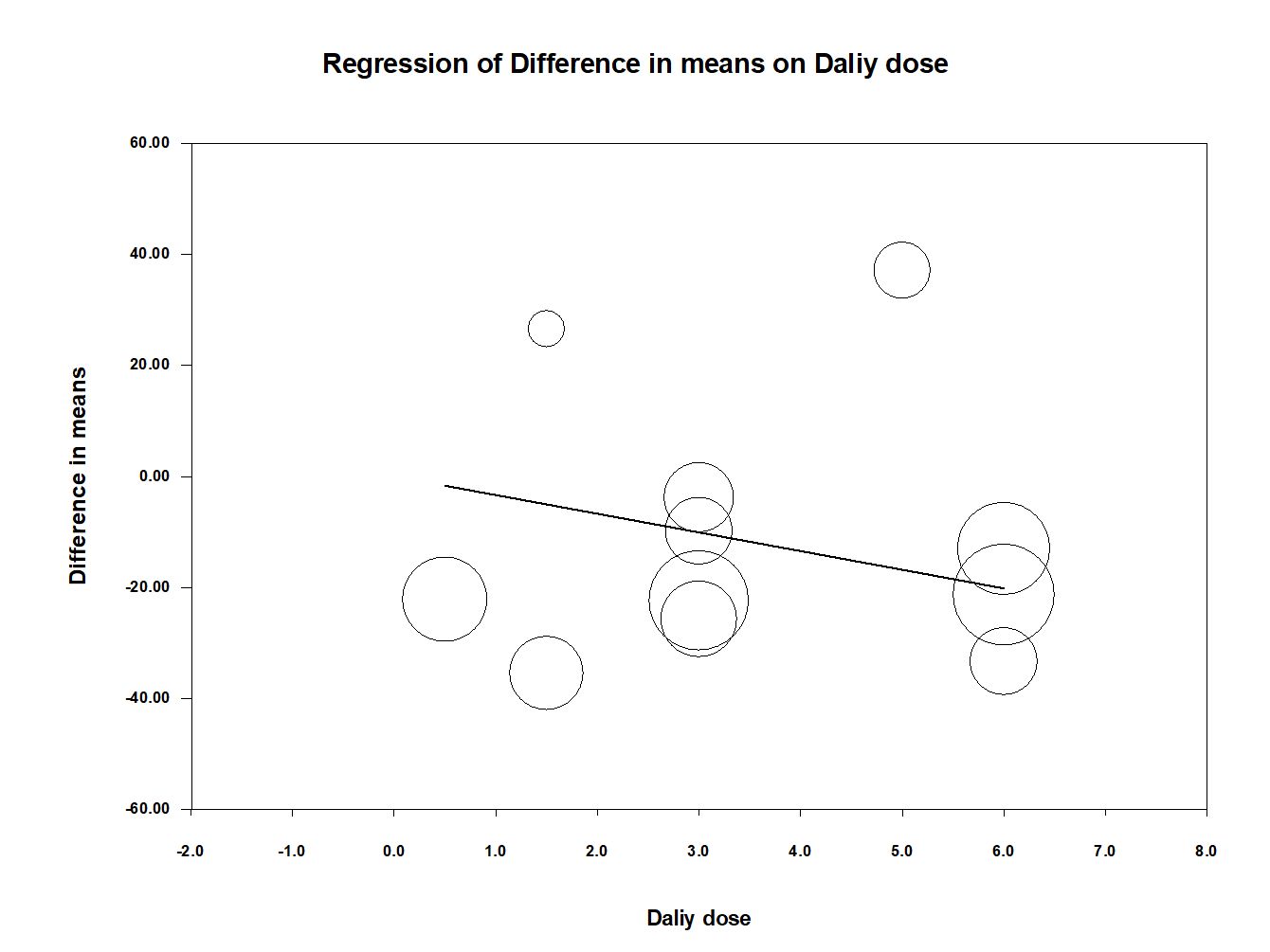


**Figure S11.** Results of sensitivity analysis using the one-study removal method to assess the impact of taurine on the overall effect size for high density lipoprotein-cholesterol

**
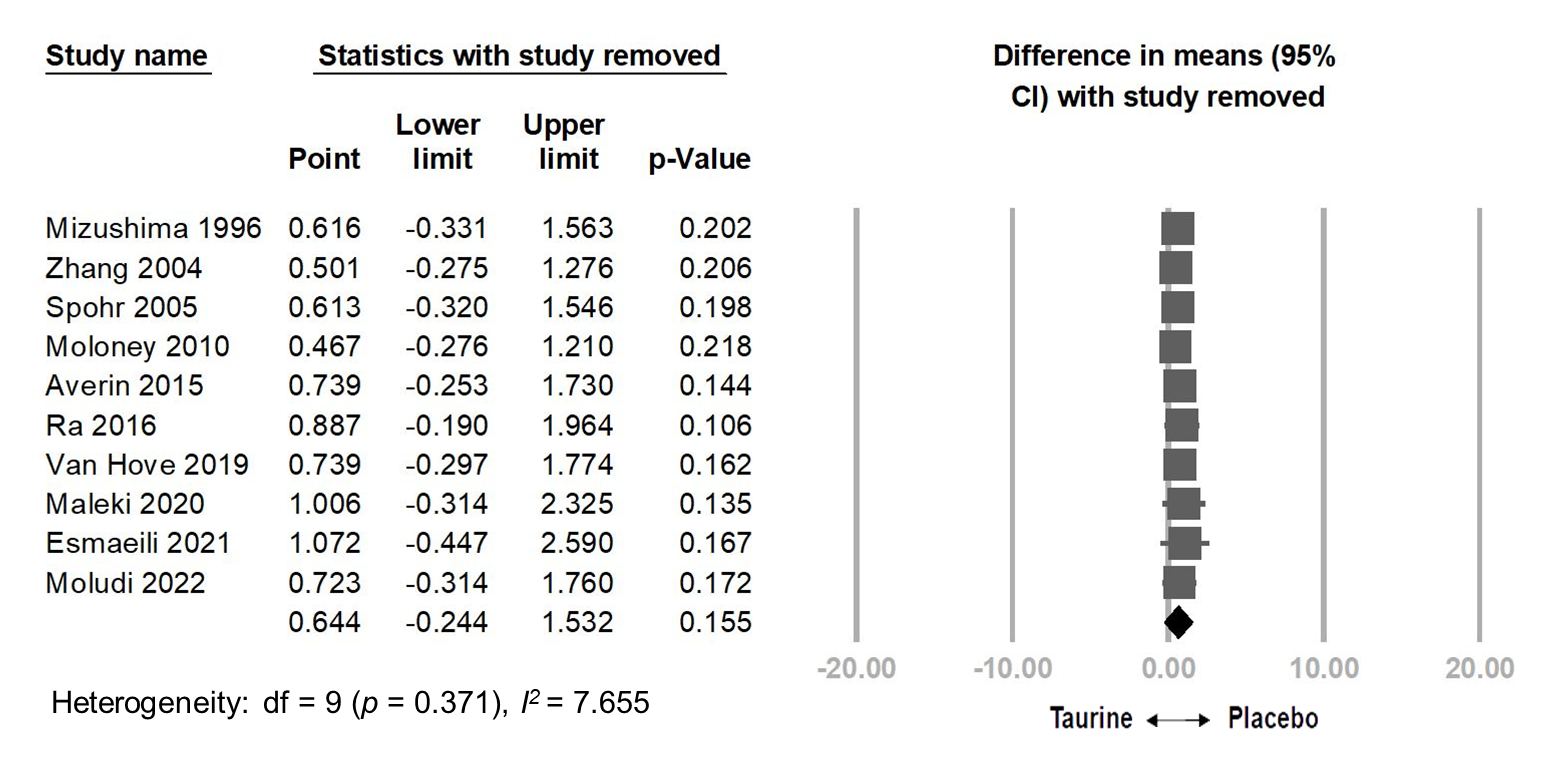
**

**Figure S12.** Meta-regression analysis showing the relationship between the total taurine dose throughout the treatment periods and high density lipoprotein-cholesterol


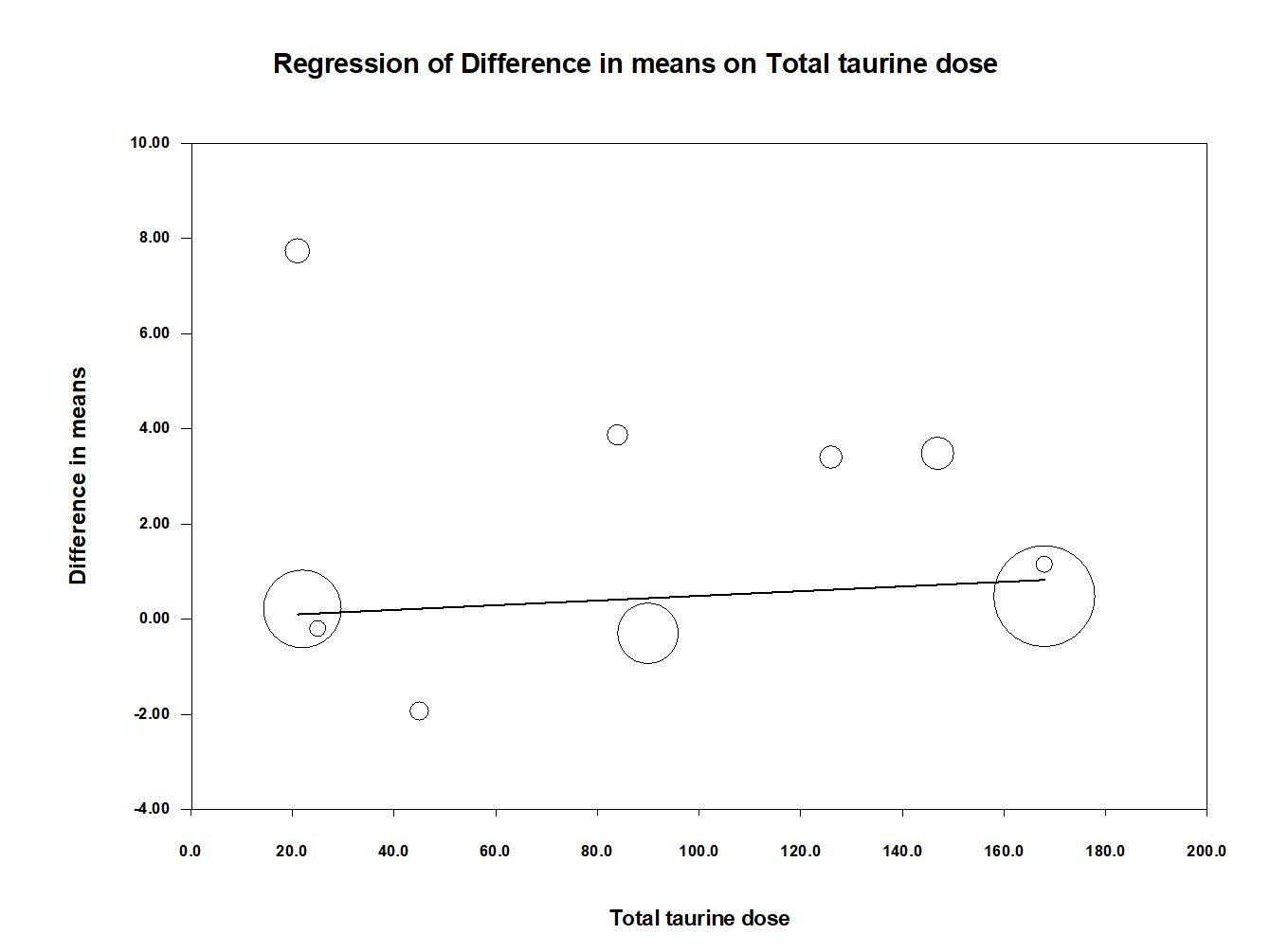


**Figure S13.** Meta-regression analysis showing the relationship between the daily dose of taurine and high density lipoprotein-cholesterol


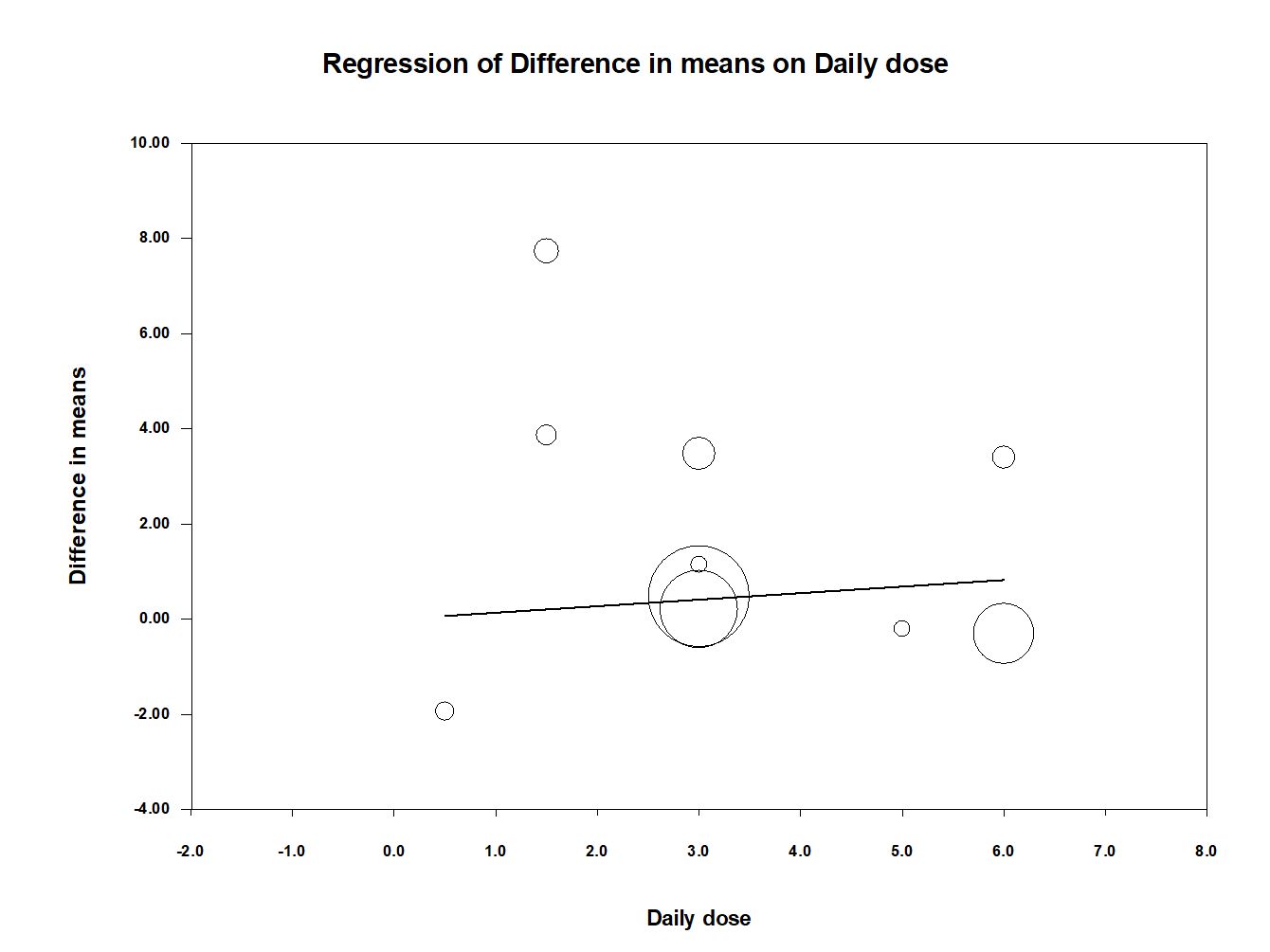


**Figure S14**. Funnel plot depicting the distribution of effect sizes for (a) systolic blood pressure (b) diastolic blood pressure across studies


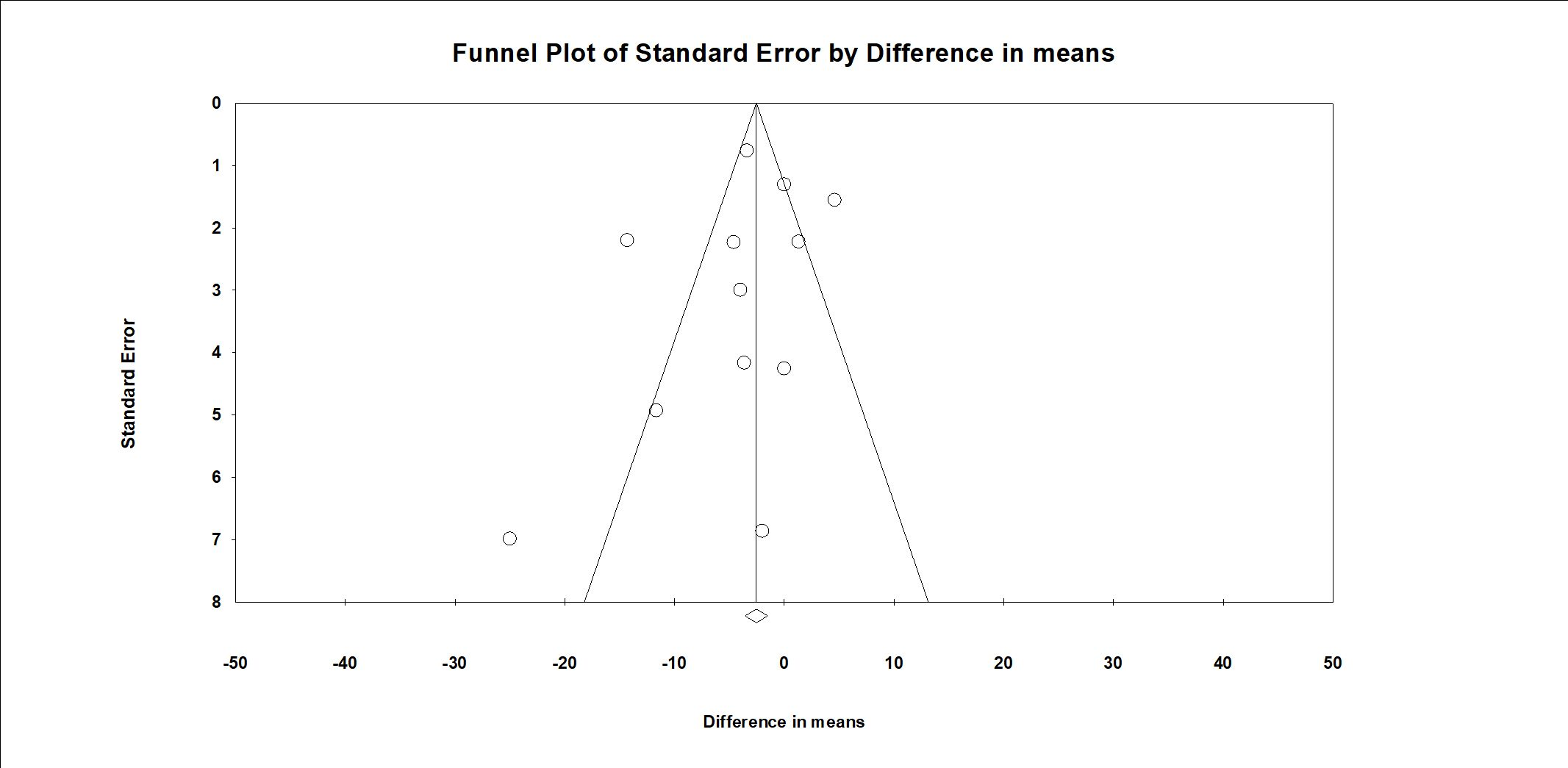


a


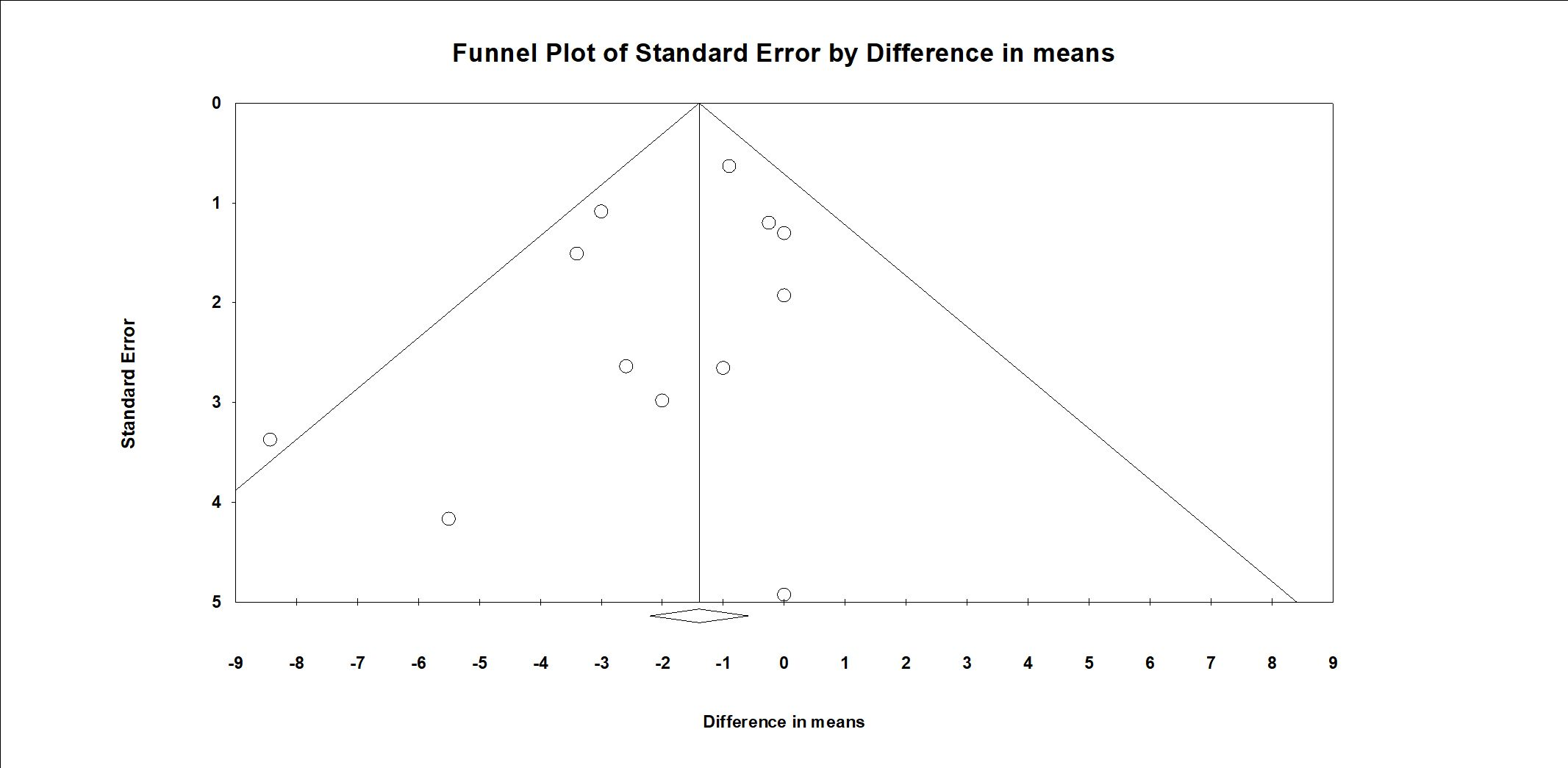


b

**Figure S15**. Funnel plot depicting the distribution of effect sizes for fasting blood glucose across studies


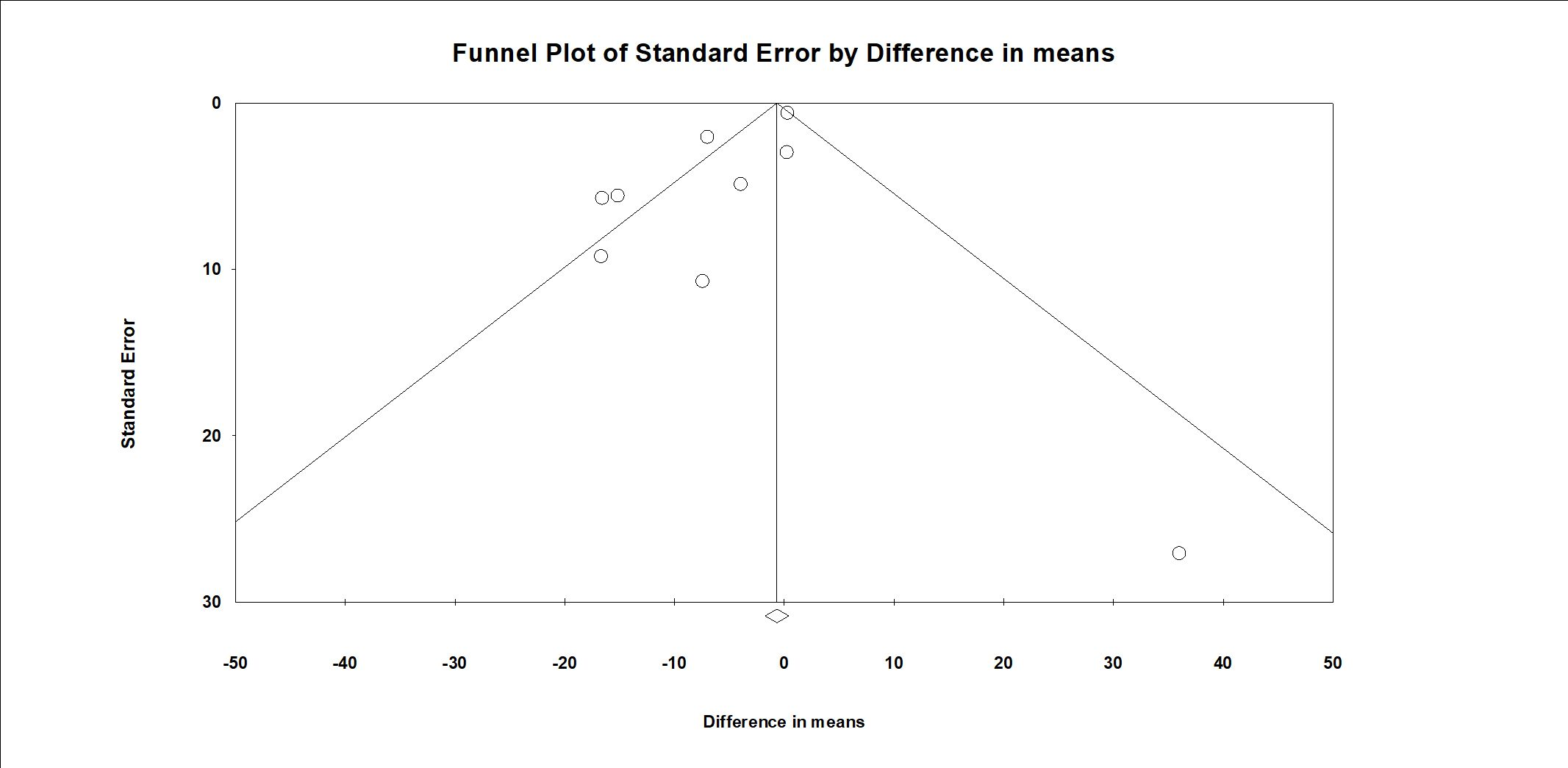


**Figure S16**. Funnel plot depicting the distribution of effect sizes for triglyceride across studies


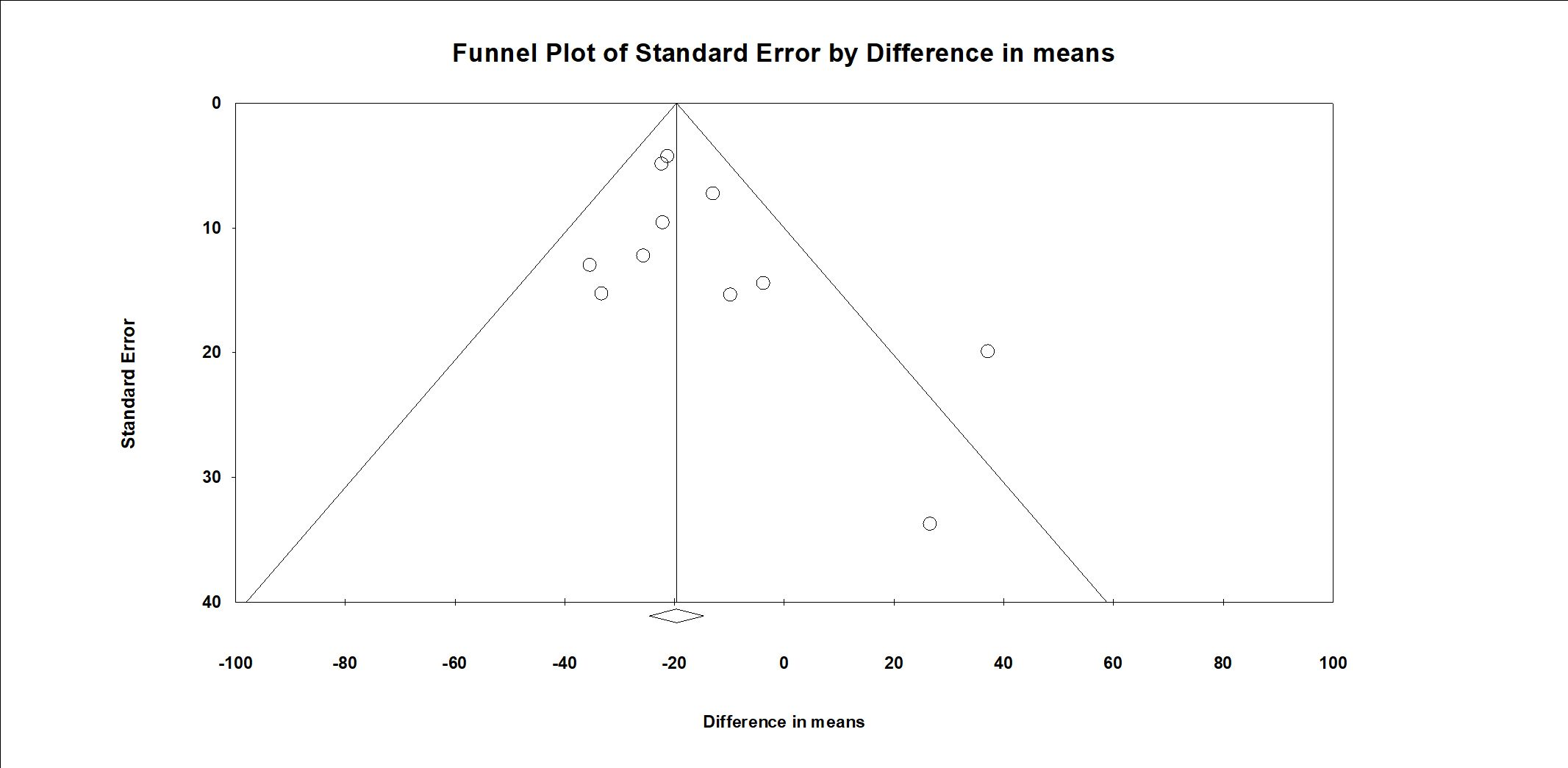


**Figure S17**. Funnel plot depicting the distribution of effect sizes for high density lipoprotein-cholesterol across studies


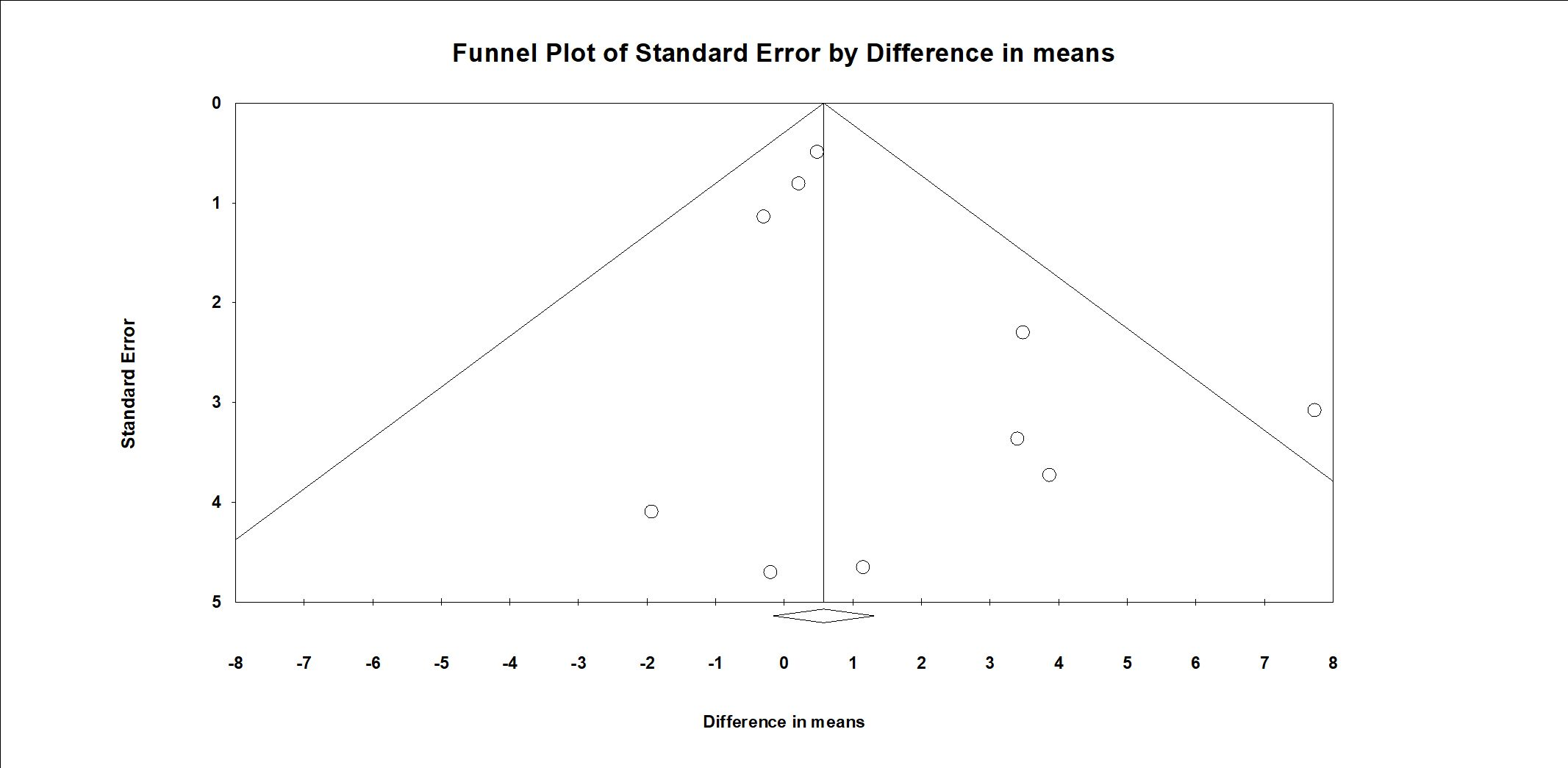


**Figure S18.** Forest plot of the overall effects of taurine on (a) body weight (BW) (b) body mass index (BMI); Results of sensitivity analysis using the one-study removal method to assess the impact of taurine on the overall effect size for (c) BW (d) BMI


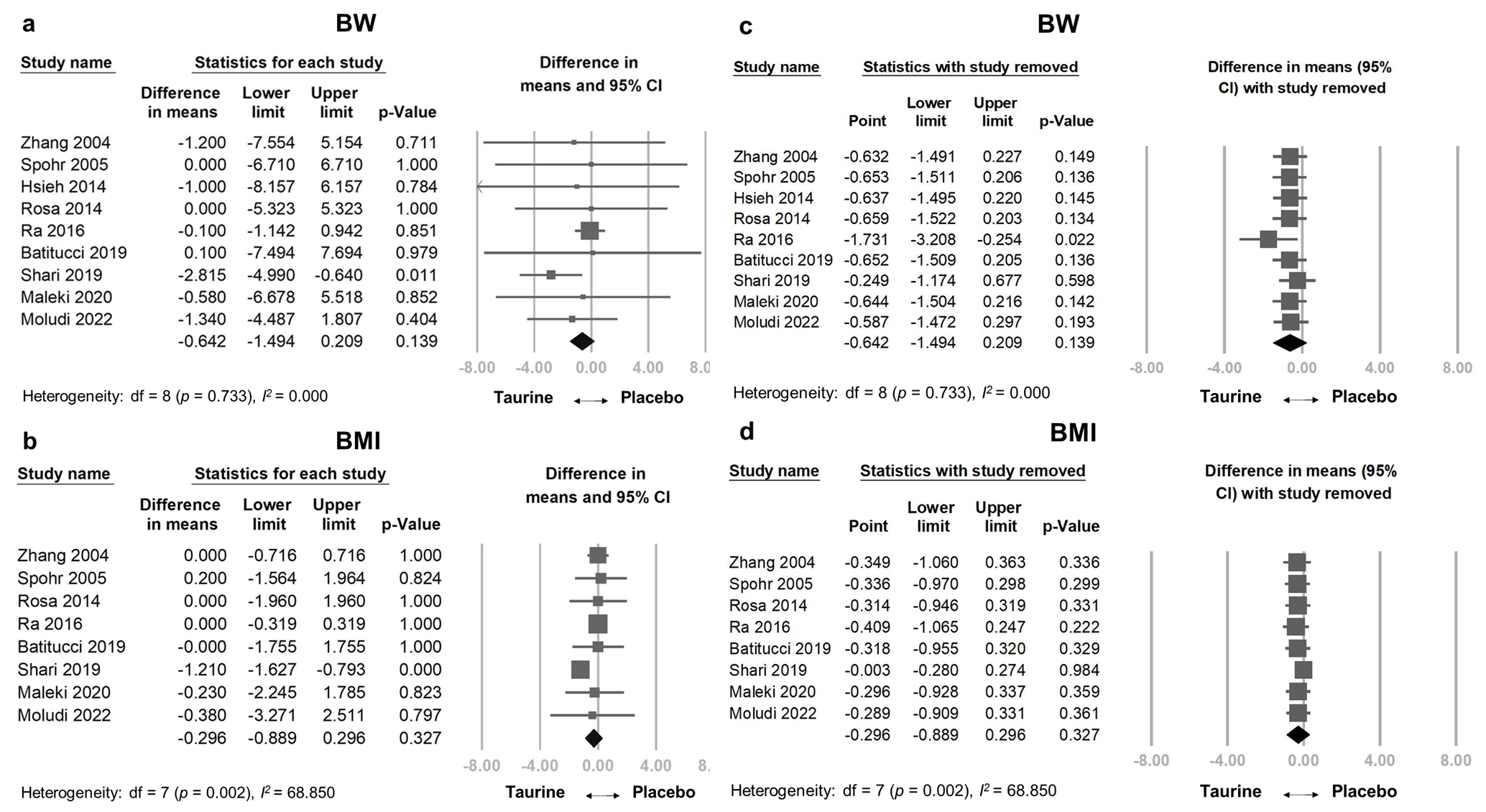


**Figure S19.** Forest plot of the overall effects of taurine on (a) lipid profiles including total cholesterol (TC) (b) low-density lipoprotein cholesterol (LDL-C); results of sensitivity analysis using the one-study removal method to assess the impact of taurine on the overall effect size for (c) TC (d) LDL-C

**
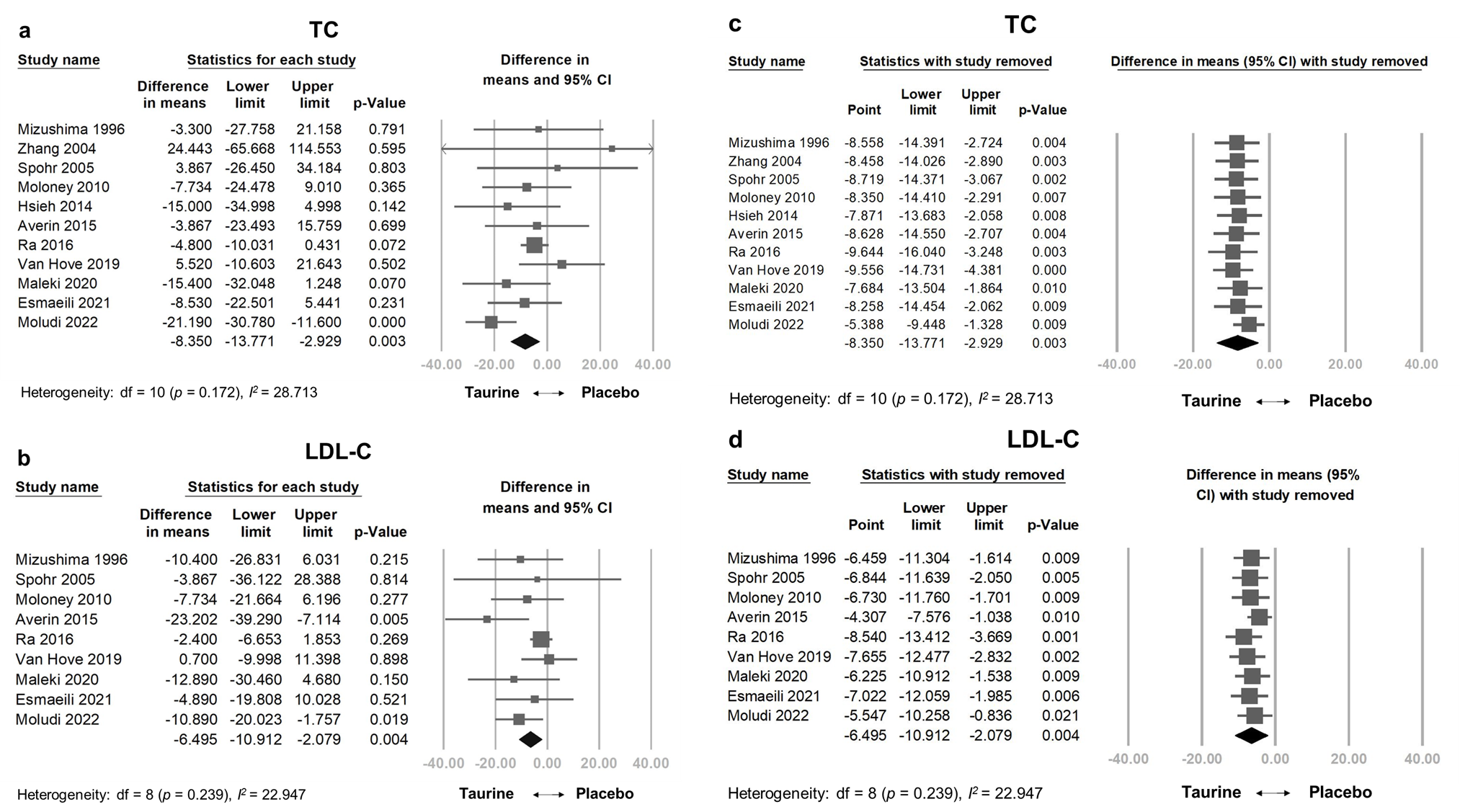
**

**Figure S20.** Forest plot of the overall effects of Taurine on (a) HbA1c (b) homeostatic model assessment (HOMA) (c) fasting insulin; results of sensitivity analysis using the one-study removal method to assess the impact of Taurine on the overall effect size for (d) HbA1c (e) homeostatic model assessment (HOMA) (f) fasting insulin


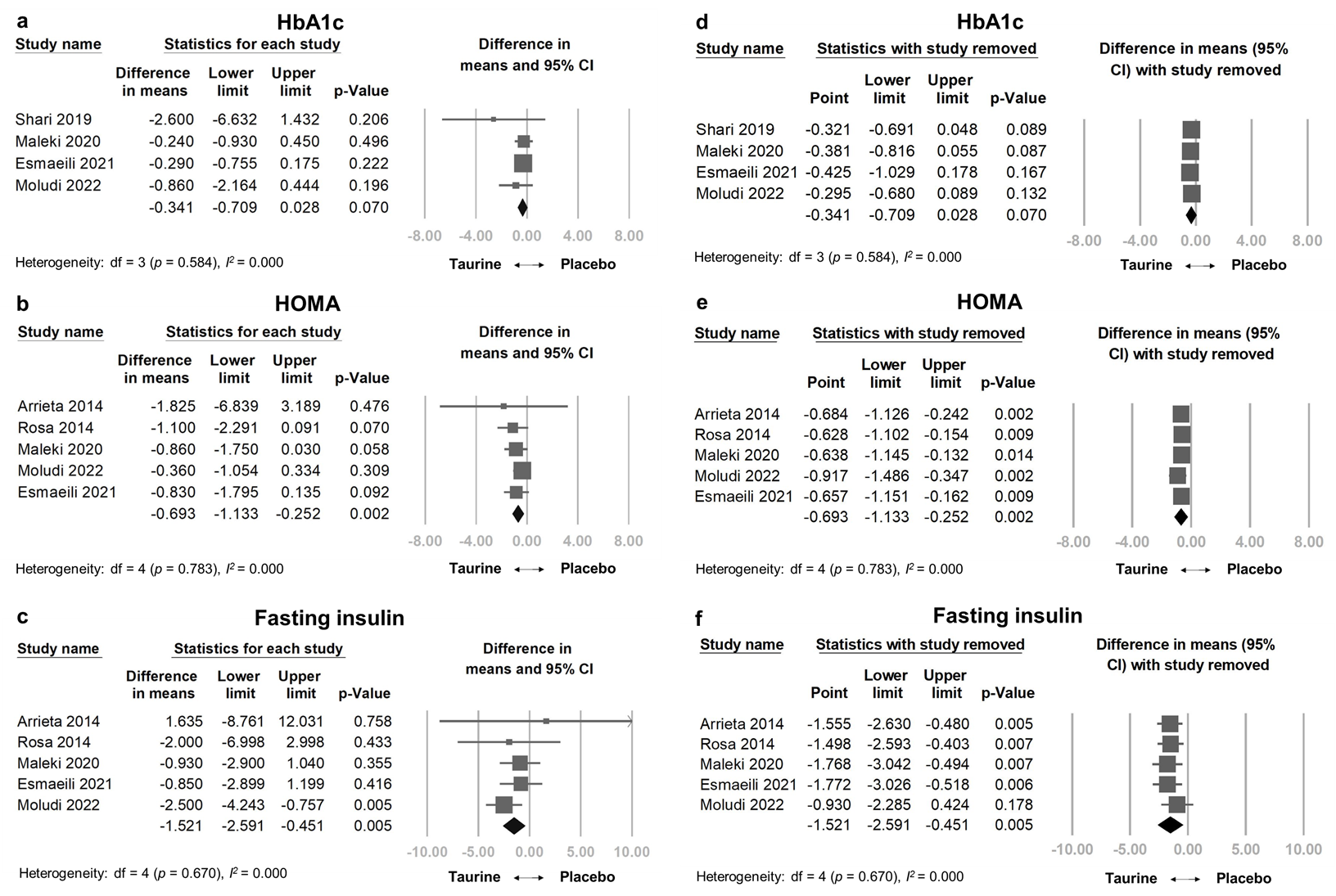


**Figure S21.** Forest plot of the treatment-associated adverse effects rates

**
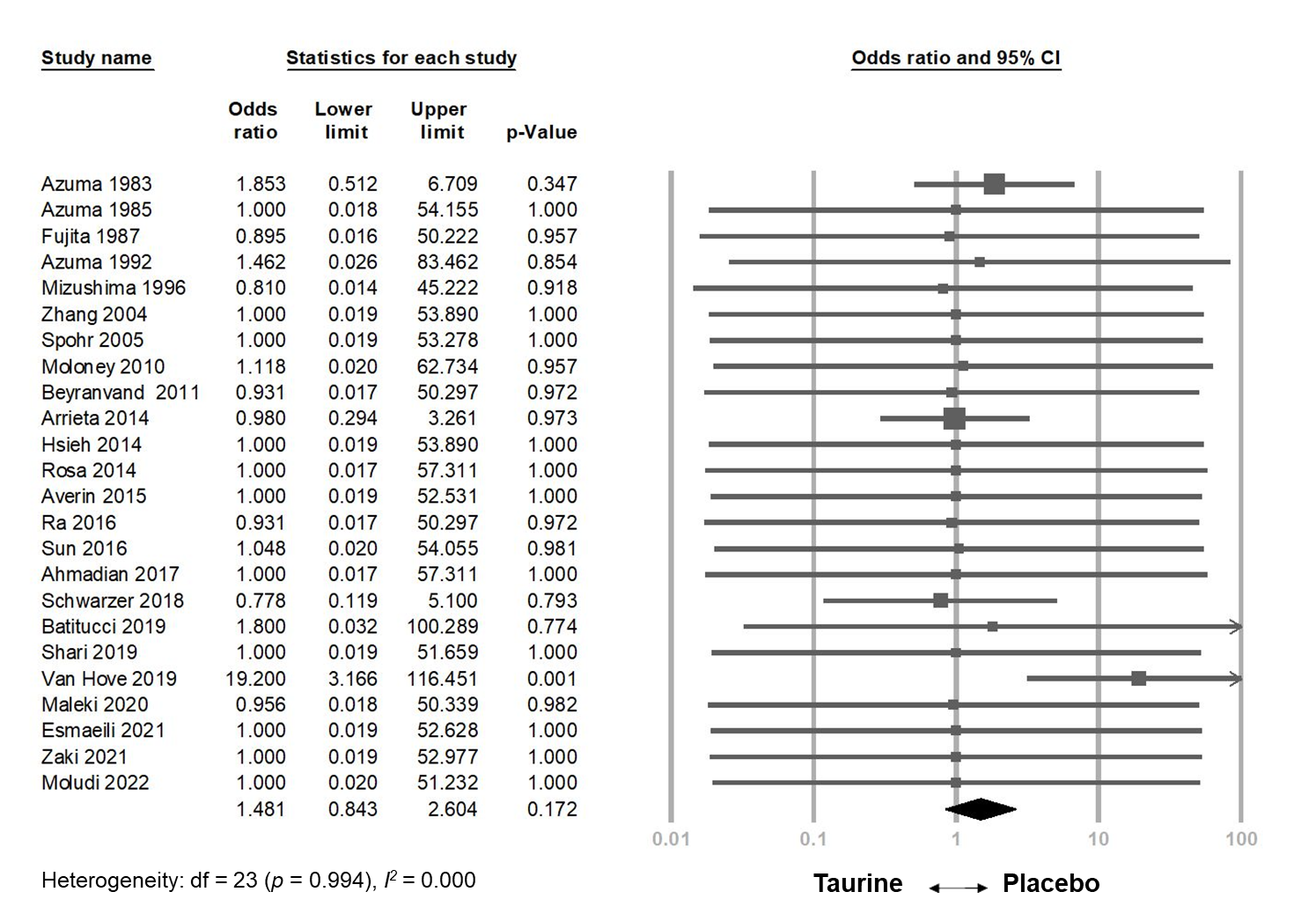
**
